# A defective splicing machinery promotes senescence through MDM4 alternative splicing

**DOI:** 10.1111/acel.14301

**Published:** 2024-08-08

**Authors:** Mathieu Deschênes, Mathieu Durand, Marc‐Alexandre Olivier, Alicia Pellerin‐Viger, Francis Rodier, Benoit Chabot

**Affiliations:** ^1^ Department of Microbiology and Infectious Diseases, Faculty of Medicine and Health Sciences Université de Sherbrooke Sherbrooke Quebec Canada; ^2^ Centre de Recherche du Centre Hospitalier de l'Université de Montréal (CRCHUM) Montréal Quebec Canada; ^3^ Institut du Cancer de Montréal Montréal Quebec Canada; ^4^ Department of Radiology, Radio‐Oncology and Nuclear Medicine Université de Montréal Montréal Quebec Canada

**Keywords:** alternative splicing, MDM4, RNA, senescence, spliceosome, splicing factors

## Abstract

Defects in the splicing machinery are implicated in various diseases, including cancer. We observed a general reduction in the expression of spliceosome components and splicing regulators in human cell lines undergoing replicative, stress‐induced, and telomere uncapping‐induced senescence. Supporting the view that defective splicing contributes to senescence, splicing inhibitors herboxidiene, and pladienolide B induced senescence in normal and cancer cell lines. Furthermore, depleting individual spliceosome components also promoted senescence. All senescence types were associated with an alternative splicing transition from the *MDM4‐FL* variant to *MDM4‐S*. The *MDM4* splicing shift was reproduced when splicing was inhibited, and spliceosome components were depleted. While decreasing the level of endogenous *MDM4* promoted senescence and cell survival independently of the *MDM4‐S* expression status, cell survival was also improved by increasing *MDM4‐S*. Overall, our work establishes that splicing defects modulate the alternative splicing of *MDM4* to promote senescence and cell survival.

## Graphical abstract

Reduced expression of spliceosome components and splicing regulators were observed in senescent cells. Splicing inhibitors and depletion of spliceosome components induced senescence, associated with an alternative splicing shift from MDM4‐FL to MDM4‐S. Decreasing MDM4‐FL promoted senescence, while increasing MDM4‐S and decreasing MDM4‐FL improved cell survival.

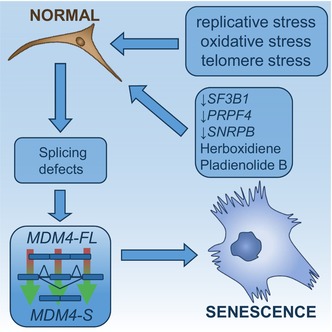

**Abbreviations**
KD: knockdown
PBS: Phosphate buffered saline
PD: population doubling
pre‐mRNA: precursor mRNA
PSI: Percent Splice‐In
RSEN: replicative senescence
SASP: senescence‐Associated Secretory Phenotype
SA‐β‐gal: senescence‐associated β‐galactosidase
SD: standard deviation
SIPS: stress‐induced premature senescence
snRNPs: small nuclear ribonucleoproteins

## 1 INTRODUCTION

Splicing is a ubiquitous process that removes introns from precursor mRNA molecules (pre‐mRNAs) (Sharp, 1994). Splicing is orchestrated by the spliceosome, a multicomponent machinery composed of five small nuclear ribonucleoproteins (snRNPs), and hundreds of proteins (Wan et al., 2020; Will & Luhrmann, 2011). In metazoans, the alternative use of splice sites is regulated by RNA binding proteins such as SR and hnRNP proteins to produce distinct protein‐coding mRNAs from a single pre‐mRNA template (Black, 2003; Blencowe, 2006; Fu & Ares, 2014; Kornblihtt et al., 2013). Ensuring the precise regulation of alternative splicing is of paramount importance, and the defective expression or activity of splicing factors often leads to various disorders, including age‐related diseases and cancer (Biamonti et al., 2019; Chabot & Shkreta, 2016; Deschênes & Chabot, 2017; Papasaikas et al., 2015; Perrone et al., 2020; Saltzman et al., 2011). For instance, oncogenic mutations in spliceosome components SF3B1 and U2AF1 result in the aberrant use of alternative branchsites (Alsafadi et al., 2016; Ilagan et al., 2015; Shiozawa et al., 2018), and cryptic splice sites (Darman et al., 2015; Kesarwani et al., 2017) that often disrupt splicing of cancer‐associated transcripts (Ilagan et al., 2015; Kesarwani et al., 2017). Overall, splicing defects ultimately lead to a failure of homeostasis, and are therefore expected to trigger a physiological response from affected cells.

Senescence is a cellular stress response characterized by the upregulation of cyclin‐dependent kinase inhibitors such as p21, p15, or p16, forcing cells into stable proliferation arrest (Campisi, 2013). It was first observed in primary cells after long periods of culturing (Hayflick, 1965; Hayflick & Moorhead, 1961). In addition to replicative senescence (RSEN), senescence is also provoked by the abnormal level of oncogenes (oncogene‐induced senescence) or by DNA damaging agents (stress‐induced premature senescence (SIPS)) (Allsopp et al., 1992; Harley et al., 1992; Serrano et al., 1997; Toussaint et al., 2000). Senescence therefore offers an important barrier against malignancies, and cancer progression will often require overcoming senescence‐associated pathways. Senescent cells release a cocktail of inflammatory factors known as the Senescence‐Associated Secretory Phenotype (SASP) that promote local tissue remodeling around senescent cells including in cancer microenvironments (Coppé et al., 2010; Faget et al., 2019; Freund et al., 2010). The SASP also promotes paracrine senescence in neighboring cancerous cells, adding another layer of defense against cancer progression (Acosta et al., 2013; Faget et al., 2019; Langhi Prata et al., 2023).

Although several links between senescence and alternative splicing have been reported (Deschênes & Chabot, 2017), how splicing regulation contributes to senescence is unclear. Splicing regulators are generally downregulated during senescence and some of them, including *SRSF3*, *SRSF7*, *hnRNP F/H*, and *YBX1*, provoke senescence when repressed (Dong et al., 2018; Hong et al., 2023; Kwon et al., 2021; Tang et al., 2013; Xiao et al., 2023; Xu et al., 2020). Likewise, compounds that reverse senescence‐associated phenotypes increase the expression of splicing regulators (Latorre et al., 2017, 2018, 2019). The splicing regulator PTBP1 positively modulates the expression of a SASP subset by promoting an exon skipping event in *EXOC7* (Georgilis et al., 2018). While our understanding of the role of spliceosome components in senescence is limited, the knockdowns (KD) of *U2AF1, XAB2*, and *PRPF19* can induce senescence (Hou et al., 2019; Yano et al., 2021; Yao et al., 2020). Additionally, the repression of *U2AF1* promote senescence in part through an intron retention event in *CPNE1* (Yao et al., 2020). Alternative splicing changes that elicit senescence were also identified in *p53* (Chen et al., 2017; Fujita et al., 2009; Tang et al., 2013) and *LMNA* (Bidault et al., 2020; Wheaton et al., 2017). Other splicing changes, such as in *POLR2A* (Hou et al., 2019) and *MDM4* (Yano et al., 2021), have been associated with senescence but the contribution of their splicing variants remain unclear.

Here, by using a collection of senescence models, we establish that a global deficit of splicing regulators and spliceosome components is a hallmark of senescence. We address the potential contribution of this deficiency by provoking senescence using splicing inhibitors and by knocking down several spliceosome components. Finally, we identify alternative splicing alterations that are shared by RSEN, SIPS, telomere‐uncapping induced senescence and senescence provoked by spliceosome‐associated defects. Among these, the alternative splicing of *MDM4* contributes to senescence via the dual effect of splice variants *MDM4‐FL* and *MDM4‐S*. Taken together, our work demonstrates that splicing defects promote senescence by modulating the alternative splicing of specific sensors like *MDM4*.

## 2 MATERIALS AND METHODS

### 2.1 Cell culture

Primary foreskin fibroblast BJ cells were kindly provided by Pr. James Smith (Baylor College of Medicine, Houston, USA), and HEK293T cells by Pr. Sherif Abou Elela (Université de Sherbrooke, Sherbrooke, Canada). HIEC‐6 cells were kindly provided by Pr. Nathalie Rivard (Université de Sherbrooke, Sherbrooke, Canada). BJ cells were cultured in αMEM (Wisent Bioproducts, catalog no. 310‐010‐CL) + 10% fetal bovine serum (FBS). WI‐38 and HEK293T were cultured in DMEM (Wisent Bioproducts, catalog no. 319‐016‐CL) + 10% FBS. HIEC‐6 were cultured in Opti‐MEM (Thermo Fisher Scientific, catalog no 31985062) supplemented with 20 mM HEPES +10 mM GlutaMAX (Thermo Fisher Scientific, catalog no. 35050061), 10 ng/mL epithelial growth factor and 4% FBS. All cell lines were incubated at 37°C in an atmosphere supplemented with 5% CO_2_. Population doublings (PD) of BJ, WI‐38 and HIEC‐6 cells were calculated using the formula LOG2 (trypsinised cells/seeded cells) + previous PD. All cell lines were trypsinised with Trypsin 0.05%/EDTA 0.53 mM (Wisent Bioproducts, catalog no. 325‐041‐EL).

### 2.2 Transfection and transduction

All transfections were performed using Lipofectamine 2000 (Thermo Fisher Scientific, catalog no. 11668019). HEK293T cells were used to generate 3rd generation lentivirus with packaging plasmids pLP1, pLP2 and envelope plasmid pLP/VSVG, kindly provided by Pr. Brendan Bell (Université de Sherbrooke, Sherbrooke, Canada). Transfer plasmid pLKO‐Tet‐On (Wee et al., 2008; Wiederschain et al., 2009) (kindly provided by Pr. Benoit Laurent (Université de Sherbrooke, Sherbrooke, Canada)) was used to generate lentivirus encoding inducible short‐hairpin RNAs (shRNA). shRNA sequences are provided in Table S3. pLenti‐6V5A (Pr. B. Bell) and pLenti‐puro (Addgene, catalog no. 39481) were used to generate lentivirus encoding specific cDNA. Lentivirus production, transduction and titers were performed according to the online “pLKO‐Tet‐On User Manual Revised” protocol found at https://media.addgene.org/data/41/67/165920fc‐af64‐11e0‐90fe‐003048dd6500.pdf. BJ cells were transduced overnight with lentiviruses using 4 μg/mL polybrene (Millipore Sigma, catalog no. H9268). Infected BJ cells were then selected using puromycin 0.5 μg/mL for pLenti‐puro and pLKO‐Tet‐On transductions, and blasticidin 5 μg/mL for pLenti‐6V5A transductions. BJ‐TIN2DN cells overexpressing H2B‐GFP fusion protein were generated as previously described (Ghadaouia et al., 2021).

### 2.3 Cellular treatments

All UV treatments were performed with 50–70 J/m^2^ using a Stratalinker 2400 (four lamps out of five were removed for precision). Cells were washed with phosphate buffered saline (PBS) and treated in 6‐well plates upon PBS removal. Herboxidiene (Focus Biomolecules, catalog no. 10–1614) and Pladienolide B (Cayman Chemical, catalog no. 16538) were dissolved in dimethyl sulfoxide (DMSO) to achieve a 1 mM stock concentration and diluted to 10 nM during treatment. All solvent controls were diluted accordingly. The expression of TetO‐dependent inserts was induced with doxycycline hyclate (Millipore Sigma, catalog no. 324385) at a concentration of 2 μg/mL. Doxycycline was dissolved in water at 2 mg/mL stock concentration. For treatments extending over 2 days, all compounds were renewed along with fresh media once every 48 h.

### 2.4 Proliferation rate assay

Cell proliferation was quantified using the Crystal Violet staining assay. In short, cells were seeded separately for each desired timepoint. The “day 0” timepoint was set at the start of treatments or 16 h after plating the cells if no treatments were involved. At each timepoint, the corresponding plate was fixed in PBS + 1% glutaraldehyde (Thermo Fisher Scientific, catalog no. O2957‐1) for 10 min at room temperature. The cells were washed with PBS once and then stored in PBS at 4°C for the remaining of the experiment (plates can be stored at 4°C for 1–2 weeks). For quantification, PBS was removed, and 0.1% Crystal Violet in H_2_O was added to each well for 30 min at room temperature. Cells were gently washed in running water and dried completely. Crystal violet was eluted with 1 mL (for 12‐well plates) of acetic acid 10% in water. The eluates were transferred in cuvettes or in 96‐well plates for absorbance analysis at 590 nm on a spectrophotometer Beckman DU800 and on a luminometer Flexstation 3 for larger dataset. Proliferation rate of BJ‐TIN2DN cells was measured using live‐imaging analysis as previously described (Ghadaouia et al., 2021).

### 2.5 Senescence‐associated β‐galactosidase assay

The assay was performed as described in Debacq‐Chainiaux et al., 2009. Briefly, cells were first washed once with PBS, then fixed with a fresh solution of 2% formaldehyde +0.2% glutaraldehyde in PBS for 4 min. Fixed cells were washed twice with PBS before adding the staining solution and incubated overnight at 37°C. Cells were then washed once with PBS followed by 3–4 washes with methanol. The plate was then dried completely and stored in the dark at room temperature. The staining solution was freshly prepared and kept in the dark as: 40 mM citric/Na‐phosphate buffer of various pH (5.7–6.0), 5 mM potassium ferrocyanide, 5 mM potassium ferricyanide, 150 mM sodium chloride, 2 mM magnesium chloride and 1 mg/mL X‐gal (Wisent Bioproducts, catalog no. 800‐145‐UG), completed in distilled water. Citric/Na‐phosphate buffer was prepared using stock solution of 100 mM citric acid and 200 mM sodium phosphate dibasic (pH 6.0 = 37 mL citric acid +63 mL sodium phosphate) and was stored at 4°C. Stock solutions for potassium ferrocyanide and potassium ferricyanide was prepared at 100 mM each and stored in the dark at 4°C. X‐gal stock solution was prepared fresh in N,N‐dimethylformamide at a concentration of 20 mg/mL and kept in the dark. Percentage of SA‐β‐Gal positive BJ‐TIN2DN cells was obtained by staining the nucleus with DAPI as previously described (Ghadaouia et al., 2021).

### 2.6 Endpoint‐RT‐PCR, qRT‐PCR, and ddRT‐PCR

RNA extractions were performed with QIAzol Lysis Reagent (Qiagen, catalog no. 79306), following manufacturer's instructions. The RNA integrity and concentration were monitored with an Agilent 2100 Bioanalyzer or with a spectrophotometer Nanodrop 2000. Reverse transcription was performed with the Transcriptor Reverse Transcriptase enzyme (Millipore Sigma, catalog no. 3531287001), random hexamers, dNTPs and 10 U of RNAse inhibitor RNAseOUT (Invitrogen, catalog no. 10777019) in reactions of 10 μL following manufacturer's instructions. For gene expression and simple alternative splicing profiling, RT‐qPCR and Endpoint PCR were used respectively and were performed by the *Plateforme RNomique de l*'*Université de Sherbrooke*, as described previously (Sohail et al., 2021). The housekeeping genes used for qPCR normalization were *L3MBTL2*, *TMEM199*, and *VAMP7*, and were selected based on their expression stability during RSEN (Hernandez‐Segura et al., 2019). For more complex alternative splicing profiling, ddRT‐PCR (droplet digital RT‐PCR) was also performed and analysed by the *Plateforme RNomique de l'Université de Sherbrooke*. Briefly, 60 ng of cDNA was mixed with 2X QX200 ddPCR EvaGreen Supermix (Bio‐Rad, catalog no. 186–4034) and 200 nM final primers concentration for 20 μL reactions. Droplets from reaction mix were generated with a Bio‐Rad QX200 droplet generator and PCR was conducted on droplets with a Bio‐Rad C1000 deep well Thermocycler. The droplets were collected and read by a Bio‐Rad QX200 Reader and the data in copies/μL, was generated by QuantaSoft from Bio‐Rad. As opposed to Endpoint‐RT‐PCR, alternative splicing variants are amplified independently. The data were converted to PSI and ΔPSI as previously described (Sohail et al., 2021). For Endpoint‐RT‐PCR products analysed on agarose gels, reverse transcription was performed with the Omniscript RT kit (Qiagen, catalog no. 205113) following manufacturer's instructions. PCR was done with 1 U of Taq polymerase with the corresponding buffer, 80 μM dNTPs, 200 nM of each primer and 10 to 100 ng of cDNA per reaction. Agarose gels were stained with RedSafe (FroggaBio, catalog no. 21141) and revealed with a Quantum ST5 from MBI lab equipment. Data from gel pictures were acquired with software ImageJ. Primer sequences are provided in Table S4.

### 2.7 Transcriptome analysis

RNA was extracted with TRIzol (Invitrogen, catalog no. 15596026) following manufacturer's instruction up until the aqueous phase separation which was carefully collected and mixed with one volume of 70% ethanol and applied to a RNeasy Mini kit column (Qiagen, catalog no. 74004). RNA integrity was assessed using an Agilent 2100 Bioanalyzer. The RNA was sent for sequencing in duplicate to the Centre for Applied Genomics at The Hospital for Sick Children in Toronto, Canada. 800 ng per sample was used as input for the NEBNext Ultra II Directional RNA Library Prep Kit for Illumina (New England Biolabs, catalog no. E7760) with the NEBNext PolyA mRNA Magnetic Isolation Module (New England Biolabs, catalog no. E7490) for library prep. 7 PCR cycles were performed and V4 chemistry was used to generate paired end reads of 2 × 126 bp. Libraries were pooled and distributed on 2 separate lanes to be sequenced on a HiSeq 2500 platform. Raw RNAseq data was trimmed using Trimmomatic (Bolger et al., 2014) and reads were aligned to the transcriptome reference genome database (RefSeq curated V109) using Bowtie2 (Langmead & Salzberg, 2012). Relative transcripts abundance was quantified using RSEM (Li & Dewey, 2011) and converted to transcripts per million (TPM). The MAJIQ software package (Norton et al., 2018) was used along with the Voila tool for alternative splicing analysis and visualization.

### 2.8 Protein analysis and antibodies

Proteins were extracted from cell pellets by sonication in Laemmli Buffer containing 5% 2‐mercaptoethanol. Protein extracts were boiled for 3 min and quantified using the Lowry method. 25 ug of proteins per sample were loaded in 10% 1:29 acrylamide gel for SDS‐PAGE. Separated proteins were transferred on a 0.45 μm Nitrocellulose membrane (Bio‐Rad, catalog no. 1620115) for Western‐Blotting. Antibodies were used against p21 (Santa‐Cruz Biotechnology, catalog no. sc‐817), p53 (Santa‐Cruz Biotechnology, catalog no. sc‐126), H2A.X (Millipore Sigma, catalog no. 05–636), GAPDH (Novus Biologicals, catalog no. NB300‐221) and Actin (Millipore Sigma, catalog no. A2066). Corresponding secondary antibodies are conjugated with horseradish peroxidase and revealed with Clarity Western ECL Substrate (Bio‐Rad, catalog no. 1705061).

### 2.9 Cytotoxicity analysis

Cytotoxicity was measured using the kit CellTox™ Green Cytotoxicity Assay (Promega, catalog no. G8741). Briefly, 5000 cells/well were seeded in 96‐well plates for 24 h. Cells were treated as described in figure legends and media was changed after treatments for fresh media containing 1× Green dye solution (1/1000 dilution). Fluorescence was measured 24 h after treatments at 485 nm_Ex_/538 nm_Em_ with a luminometer Flexstation 3. Fluorescence was taken again for normalization purposes after cells were lysed with the lysis buffer provided with the CellTox™ kit and by following manufacturer's instructions.

### 2.10 Statistics

Values expressed as ratios are presented as geometric means with geometric SD, whereas linear values are presented as arithmetic means with arithmetic SD. Means were compared using Student's *t*‐test and geometric means were log2 transformed before conducting *t*‐tests. Equality of variance was tested using Fisher's *F*‐test prior to means comparison.

## 3 RESULTS

### 3.1 Senescence is characterized by a global downregulation of splicing factors and alternative splicing changes

For this study, the primary human diploid fibroblasts BJ, IMR‐90 and WI‐38 of advanced PD were used as models for RSEN. BJ, WI‐38 and colon cancer HCT116 cells treated with hydrogen peroxide were used as models for SIPS. Senescence was also achieved in BJ cells via the regulated expression of a dominant negative version of the telomere‐associated protein TIN2 (TIN2DN) causing telomere uncapping (Ghadaouia et al., 2021).

**FIGURE 1:**
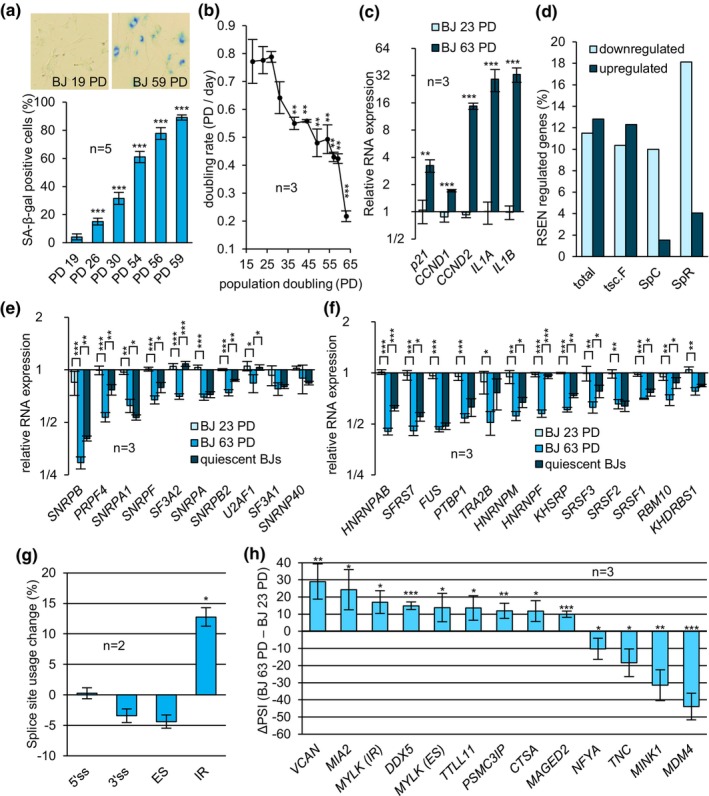
Replicative senescence is associated with a global downregulation of splicing factors. (a) BJ cells were stained with the X‐gal chromogenic substrate using the senescence‐associated β‐Galactosidase (SA‐β‐Gal) method. Positive and negative cells were individually counted in 5 randomly picked pictures for each population. Samples were statistically compared to PD 19. (b) The doubling rate of BJ cells at various PDs was quantified using the Crystal Violet staining assay. Each population was cultured for 4 days before data acquisition. The doubling rate is defined as the average amount of doublings per day achieved between day 0 and day 4 (PD/day). Each sample was statistically compared to PD 19. (c) Relative expression of senescence RNA markers in BJ cells was quantified with qRT‐PCR where values for low PD BJ cells were set to 1.0. Data from higher PD were statistically compared to data from lower PD. (d) RNAseq was performed on duplicate sets of BJ cells at PD 18 and 63. Transcripts were considered repressed or overexpressed when *log2 of senescent TPM/young TPM were* < −*0.5* or *>0.5*, respectively. Only transcripts with TPM >5 in at least one sample were considered. (total = all transcripts; tsc.F = transcription factors; SpC = spliceosome components; SpR = splicing regulators). (e, f) Relative RNA expression of spliceosome components (e) and splicing regulators (f) was assessed by qRT‐PCR. Values obtained from BJ cells of low PD were set to 1.0. (g) Percent Spliced‐In (PSI) of individual alternative splicing events and splicing modes were obtained by the *Plateforme RNomique de l'Université de Sherbrooke*. Only events with |ΔPSI| >5% were considered. For each splicing mode, PSIs (long variant)/(long + short) × 100 were multiplied by the respective gene expression level (TPM) and their sum from duplicates generated an abundance index normalized in percent change compared to low PD BJ cells. (5'ss = 5′ alternative splice site; 3'ss = 3′ alternative splice site; ES = exon skipping; IR = intron retention). (h) Alternative splicing events were validated by endpoint RT‐PCR. ΔPSI is defined as the difference between the PSI from test group and the control group. Statistical analysis was performed on PSI only. (*MYLK (IR) =* intron retention event in *MYLK*; *MYLK* (*ES) =* novel exon skipping event in *MYLK*). Quiescent low PD BJ cells were generated by contact inhibition (100% confluency). Means were compared using Student's *t*‐test. *p‐value <0.05, **p‐value <0.01, ***p‐value <0.001.

**FIGURE 2:**
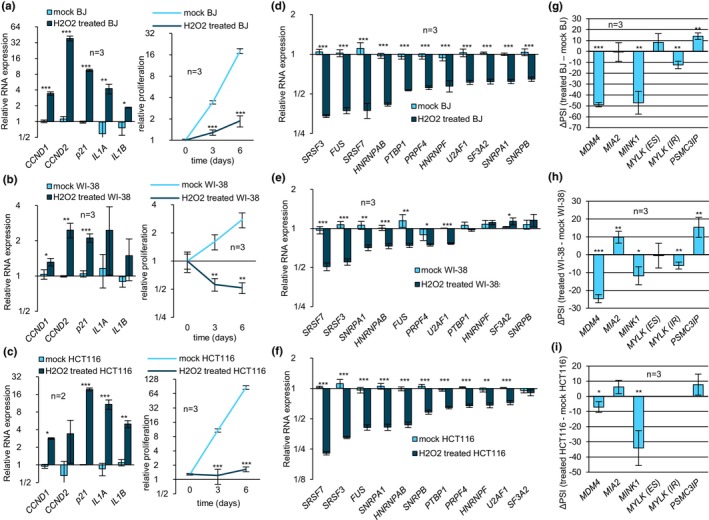
Stress‐induced premature senescence is associated with a decrease in the expression of splicing factors and changes in alternative splicing. (a–c) Relative expression of senescence RNA markers (left) in BJ (a), WI‐38 (b) and HCT116 (c) cells was quantified by qRT‐PCR where mock‐treated samples were set to 1.0. Proliferation rate (right) was quantified using the Crystal Violet staining assay. Values obtained from cells treated with hydrogen peroxide were statistically compared to mock values of corresponding timepoints. (d–f) Relative expression of splicing factors in BJ (d), WI‐38 (e) and HCT116 (f) cells was quantified with qRT‐PCR where mock samples were set to 1.0. (g–i) Alternative splicing events in BJ (g), WI‐38 (h) and HCT116 (i) cells were quantified by endpoint RT‐PCR to obtain ΔPSI values. Statistical analysis was performed on PSI only. BJ (top panels), WI‐38 (middle panels) and HCT116 (bottom panels) cells were treated with hydrogen peroxide and compared to mock‐treated samples. Means were compared using Student's t‐test. *p‐value <0.05, **p‐value <0.01, ***p‐value <0.001.

To validate RSEN, BJ cells of increasing PD were tested for senescence‐associated β‐galactosidase activity (SA‐β‐gal), proliferation rate and expression of senescence‐associated RNA biomarkers. Cells that had undergone more PD displayed both an increase in SA‐β‐gal activity and a progressive decrease in doubling rate, consistent with the onset of senescence (Figure 1a,b). In addition, RNA expression of senescence markers *p21*, *CCND1*, *CCND2*, *IL1A*, and *IL1B* increased significantly in BJ cells of higher PD (Figure 1c). In IMR‐90 cells of higher PD and slower proliferation rate, *CCND2*, and *p21* were significantly overexpressed, whereas WI‐38 cells of higher PD overexpressed *p21, IL1A*, and *IL1B* (Figure S1a,b). A partial association of senescence markers in IMR‐90 and WI‐38 cells is likely due to the smaller difference in PDs between low and higher PD populations compared to BJ cells. Peroxide‐induced SIPS in BJ cells was confirmed by an increase in all senescent markers and a significant drop in proliferation (Figure 2a). Peroxide‐treated WI‐38 and HCT116 cells also displayed proliferation arrest but only *CCND1* and *p21* were commonly upregulated (Figure 2b,c). Finally, senescence in TIN2DN‐induced BJ cells was confirmed by the increase in SA‐β‐gal activity, proliferation arrest, and an elevation in the expression of *CCND1, CCND2, IL1A*, and *IL1B* (Figure S2a–c).

To investigate changes in expression and alternative splicing, the polyadenylated transcriptome of low PDs and RSEN BJ cells was sequenced. Our RNAseq analysis indicated that an equivalent number of genes were repressed and upregulated during RSEN (Figure 1d; see “total”). However, splicing‐related genes were preferentially repressed: 18% of genes encoding splicing regulators were downregulated and only 4% of them were upregulated (Table S1 and Figure 1d, see “SpR”). Genes encoding spliceosome components displayed a similar trend where nearly 10% were repressed while 1.5% were upregulated (Table S1 and Figure 1d; see “SC”). In comparison, transcription factor‐encoding genes displayed less than a 2% difference between upregulated and repressed genes (Table S1 and Figure 1d, see “tsc.f”). Expression changes of selected splicing factors were validated by qRT‐PCR assays with the addition of quiescent early passage cells to identify alterations associated with proliferation arrest (Figure 1e,f). For most factors, the drop in expression was significantly greater in senescent cells than in nonproliferating cells. For instance, the downregulation of spliceosome components *PRPF4, SF3A2* and splicing regulators *HNRNPAB* and *hnRNPF* showed a strong association with RSEN (*p* < 0.001) when compared to quiescent cells. In contrast, the decreased expression of spliceosome components *SNRPA1*, *SNRPA*, and splicing regulators *FUS, PTBP1*, and *SRSF2* was similarly associated with senescent and quiescent cells. Notably, splicing factors *SRSF7* and *U2AF1* were downregulated in RSEN BJ, WI‐38 and IMR‐90 cells (Figure S1c, d). Splicing factors whose expression dropped in senescent BJ cells were also strongly reduced in SIPS BJ cells (*p* < 0.001) (Figure 2d). Most but not all splicing factors tested were similarly downregulated in WI‐38 and HCT116 cells undergoing SIPS (Figure 2e,f). Spliceosome components *U2AF1*, *PRPF4*, and *SNRPA1*, as well as splicing regulators *SRSF7*, *SRSF3*, *HNRNPAB*, and *FUS* were downregulated in all SIPS models. Finally, TIN2DN‐induced senescent cells also displayed a significant drop in the expression of spliceosome components *SNRPA1*, *SNRPF*, *PTBP1*, and splicing regulators *SRSF1*, SRSF2, *SRSF3, TRA2B*, and *HNRNPM* (Figure S2d,e). Our results reveal a consistent drop in the expression of splicing regulators and spliceosome components across independent primary cell cultures undergoing distinct modes of senescence. Interestingly, *SRSF7* and *U2AF1* were systematically downregulated in all our senescence models. Given that their KDs are known to provoke senescence (Kwon et al., 2021; Yao et al., 2020), the downregulation of *SRSF7* and *U2AF1* along with several other splicing regulators could play an important role in reinforcing the senescence state.

As changes in the expression of splicing factors will likely impact alternative splicing, we searched for alternative splicing changes in BJ cells undergoing senescence. RNAseq analysis revealed a 12.8% increase in retained introns but no significant change in skipped exons or alternative 5′ and 3′ splice site usage during RSEN (Figure 1g). We used RT‐PCR and ddPCR to validate a set of 13 events, including events with |∆PSI| >20 in *VCAN*, *MIA2*, *MINK1*, and *MDM4* (Figure 1h). Although expression level of *VCAN* (Yan et al., 2019), *DDX5* (McCann et al., 2023), *NFYA* (Matuoka & Chen, 2000), *TNC* (Gremlich et al., 2021), *MINK1* (Nicke et al., 2005), and *MDM4* (Mejía‐Hernández et al., 2022) had already been linked to senescence and aging, the role of their splicing variants in this context has remained largely unexplored. Additionally, *PSMC3IP* alternative splicing was altered in all RSEN models as well as in BJ and WI‐38 cells undergoing SIPS (Figure S1e,f). *MINK1* alternative splicing was consistently altered in all SIPS models of senescence (Figure 2g–i). Lastly, *MDM4* alternative splicing had the strongest change in RSEN BJ and IMR‐90 cells (Figure 1h,e), and was shifted in all SIPS models as well as in TIN2DN‐induced senescent BJ cells (Figure 2g–i and S2f). A significant change in the alternative splicing of *MIA2* and *MINK1* was also detected in TIN2DN‐induced senescent cells (Figure S2f).

Overall, our results indicate that senescence is associated with a preferential drop in the expression of spliceosome components and splicing regulators. This change is accompanied by a significant increase in intron retention events, which may indicate reduced splicing activity (AlShareef et al., 2017; Kashyap et al., 2015). Additionally, several alternative splicing alterations occurred in genes whose expression was previously associated with senescence.

### 3.2 Splicing inhibition induces senescence and senescence‐associated alternative splicing

The senescence‐associated drop in the expression of spliceosome components together with the increase in retained introns suggest that splicing inhibition is occurring in senescent cells. We tested the impact of disrupting splicing by using the splicing inhibitors Herboxidiene and Pladienolide B. Herboxidiene and Pladienolide B disrupt the SF3b complex of the U2 snRNP by binding to SF3B1 (AlShareef et al., 2017; Aouida et al., 2016; Hasegawa et al., 2011; Kashyap et al., 2015; Kumar et al., 2022). A viability assay was preliminarily performed on BJ cells to determine that 10 nM was the lowest concentration that inhibits proliferation with limited impact on cell death. In addition to BJ, WI‐38, and IMR‐90 cells, we included in this analysis the primary human intestinal epithelial cell line HIEC‐6.

**FIGURE 3:**
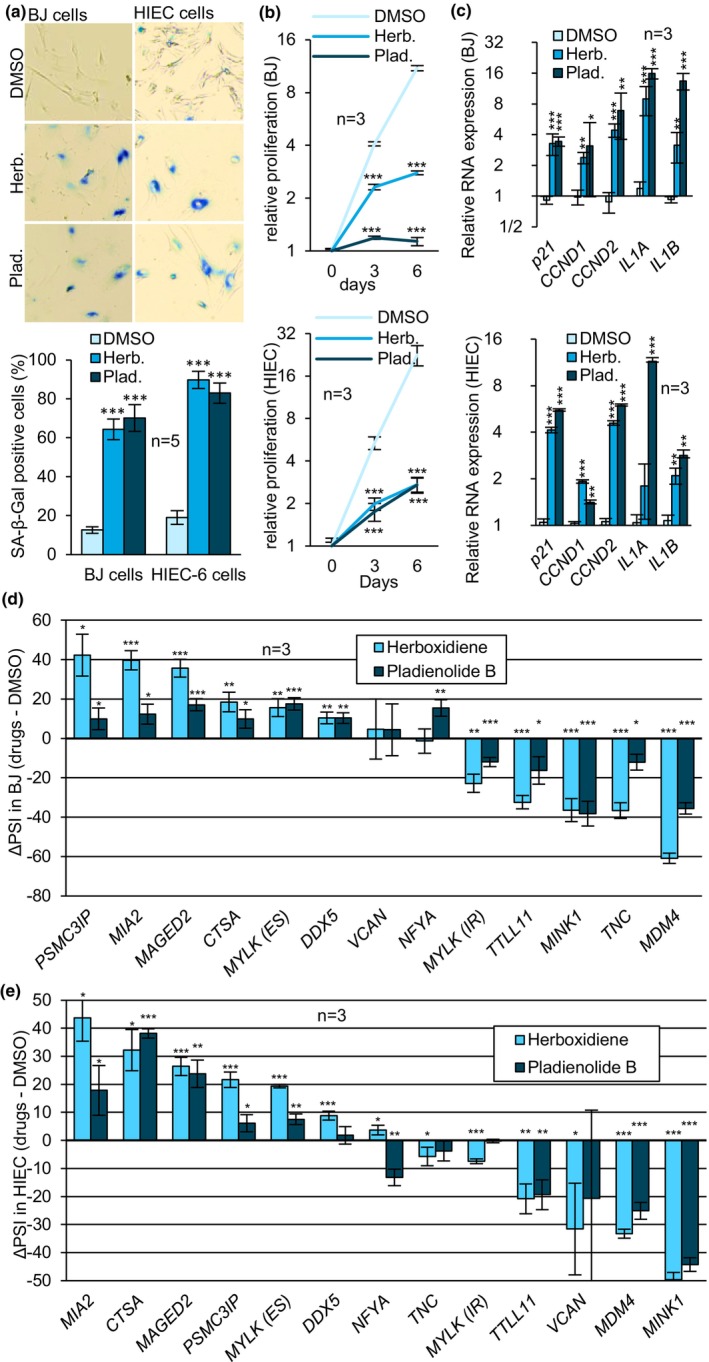
Splicing inhibition induces senescence. (a) BJ (left) and HIEC (right) cells were stained using the SA‐β‐Gal method. (b) Proliferation rate of BJ (top) and HIEC (bottom) cells was quantified using the Crystal Violet staining assay. Each sample was statistically compared with the DMSO sample at the corresponding timepoint. (c) Relative expression of senescence RNA markers in BJ (top) and HIEC (bottom) cells was quantified with qRT‐PCR where DMSO samples were set to 1.0. (d, e) Alternative splicing events were quantified by endpoint RT‐PCR in BJ (d) and HIEC cells (e). Statistical analysis was performed on PSI only. All treatments with Herboxidiene (Herb.) and Pladienolide B (Plad.) were performed at concentrations between 5 and 10 nM for a 6‐day duration (10 nM: BJ, IMR‐90, WI‐38. 5 nM: HIEC, HCT116). Samples were statistically compared to DMSO‐treated cells. Means were compared using Student's t‐test. *p‐value <0.05, **p‐value <0.01, ***p‐value <0.001.

Following a 6‐day treatment at 10 nM, Herboxidiene and Pladienolide B drastically increased SA‐β‐gal activity in both BJ and HIEC‐6 cells (*p*‐value <0.001) (Figure 3a,b). Cell proliferation was significantly reduced, and cells displayed a senescence‐like flattened and enlarged morphology. Splicing inhibitors also increased the expression of all senescence markers in both BJ and HIEC‐6 cells, except for *IL1A* in Herboxidiene‐treated HIEC‐6 cells (Figure 3c). Herboxidiene provoked a proliferation arrest in WI‐38 cells and increased the expression of all senescence markers (Figure S3a). At the concentration used, Herboxidiene did not affect the proliferation of IMR‐90 cells, but elicited significant increases in *p21*, *CCND2*, and *IL1B* (Figure S3b).

The depletion of splicing regulators and a treatment with Pladienolide B can lead to genomic instability and DNA damage (Pederiva et al., 2016; Shkreta & Chabot, 2015), and this could potentially elicit senescence in a splicing‐independent manner. To test whether the splicing inhibitors causes DNA damage, we monitored the phosphorylation of H2A.X (γH2A.X) and the abundance of p53. After a 6‐day treatment with Herboxidiene and Pladienolide B, γH2A.X and p53 remained at a level comparable to DMSO treatment (Figure S4a). As a positive control, 70 J/m^2^ of UV light provoked a marked increase in both γH2A.X and p53. This indicates that under these conditions, Herboxidiene and Pladienolide B do not elicit a robust DNA damage response. Intriguingly, the increase in p21 was not associated with a corresponding increase in p53, suggesting that the senescence induced by the drugs may be p53‐independent. To investigate this further, BJ cells were treated with both Herboxidiene and the p53 inhibitor Pifithrin‐ɑ (PFTɑ). PFTɑ did not prevent the senescence mediated by Herboxidiene (Figure S4b). Next, Herboxidiene was used to induce senescence in wild type (HCT116 p53^+/+^) and p53‐depleted HCT116 cells (HCT116 p53^−/−^). While Herboxidiene reduced proliferation in both cell lines, no discernible difference was observed between treated HCT116 p53^+/+^ and HCT116 p53^−/−^ cells (Figure S4c). Although the levels of *p21* and *IL1A* were lower in treated HCT116 p53^−/−^ compared to treated HCT116 p53^+/+^, both cell lines exhibited a significant increase in *p21*, *IL1A* and *IL1B* levels compared to their DMSO‐treated counterparts (Figure S4d). Herboxidiene‐associated alternative splicing alterations were unaffected by the lack of p53 (Figure S4e). Overall, these results suggest that splicing inhibition provokes senescence in a p53 and DNA damage‐independent manner.

We also interrogated the 13 alternative splicing events previously associated with RSEN, in BJ and HIEC cells treated with both Herboxidiene and Pladienolide B. In BJ cells, nine were significantly shifted (*PSMC3IP, MIA2, MAGED2, CTSA, MYLK (ES), DDX5, MINK1, TNC*, and *MDM4*) while seven shifted in HIEC‐6 cells (*PSMC3IP, MIA2, MAGED2, CTSA, MYLK(ES), MINK1*, and *MDM4*) (Figure 3d,e). As for WI‐38 and IMR‐90 cells treated with Herboxidiene, the alternative splicing of *MDM4, MINK1* and *PSMC3IP* was consistent with RSEN profiles (Figure S3c,d). While *VCAN* splicing was not affected by the drugs in BJ and HIEC‐6 cells, *NFYA*, *MYLK* (IR), and *TTLL11* splicing responded in the opposite way relative to RSEN. In HIEC‐6 cells, *DDX5*, and *TNC* splicing was altered only in response to Herboxidiene. Finally, the expression of all senescence markers, except p21, are significantly increased in BJ cells after a 48 h exposure to Herboxidiene (Figure S3e). Conversely, splicing shifts in *MDM4, MIA, MINK1*, and *PSMC3IP* occurred at the same time or prior to the onset of senescence markers (Figure S3f), suggesting a direct response to splicing inhibition rather than a response to the senescence program.

Taken together, our analysis shows that subtoxic concentrations of the splicing inhibitors Herboxidiene and Pladienolide B induce senescence in several cellular models. In addition, several alternative splicing changes detected during RSEN also occurred in cells treated with splicing inhibitors. Our results therefore suggests that the senescence‐associated downregulation of spliceosome components contributes to senescence by inhibiting splicing activity.

### 3.3 Defective expression of spliceosome components promotes senescence

**FIGURE 4:**
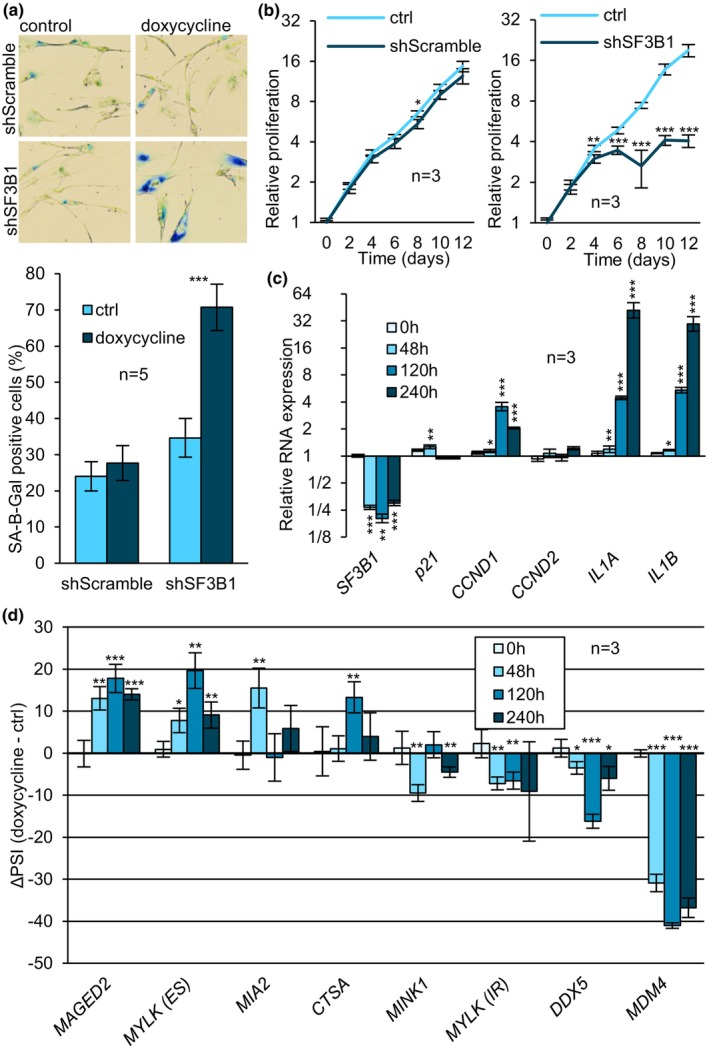
A deficit in *SF3B1* induces senescence. (a) BJ cells were stained using the SA‐β‐Gal method. (b) Proliferation rate was measured with the Crystal Violet staining assay. The values obtained for each sample were statistically compared with control group values at corresponding timepoints. (c) Relative expression of senescence RNA markers and shRNA targets was quantified with qRT‐PCR where samples taken at *t* = 0 were set to 1.0. Data for individual transcript were statistically compared to t = 0. (d) Alternative splicing events were quantified by endpoint RT‐PCR. Statistical analysis was performed on PSI only. Doxycycline induction of shRNAs targeting spliceosome components was performed for a 10‐day duration, unless indicated otherwise. Means were compared using Student's t‐test. *p‐value <0.05, **p‐value <0.01, ***p‐value <0.001.

To confirm that spliceosome defects directly contribute to senescence, we carried out the inducible KD of various spliceosome components in BJ cells. Compared to non‐induced cells or to cells induced to express a scrambled shRNA, the KD of *SF3B1* significantly increased SA‐β‐gal activity, promoted drastic proliferation arrest (*p*‐value >0.001) accompanied with morphological changes (Figure 4a,b). The expression of RNA senescence markers progressively increased during a 10 day‐induction period in shSF3B1‐expressing cells while no change was observed in control cells expressing a scrambled shRNA (Figure 4c and Figure S5a). While the KD of *SF3B1* provoked a weak but transient increase in *p21*, the expression of *IL1A* and *IL1B* increased to a level exceeding that observed during RSEN and senescence induced by splicing inhibitors (Figure 4c, see Figures 1c and 3c). In contrast to the drugs however, *CCND2* expression was not upregulated in response to the KD. Thus, while the repression of *SF3B1* promotes senescence, it does not fully reproduce the effects of splicing inhibitors.

In addition to *SF3B1*, BJ cells inducible for shRNAs targeting spliceosome components *SNRNP70, PRPF8, PRPF4* and *SNRPB* were also generated (Figure S5b). After 10 days of induction, all KDs displayed a senescence‐like morphology and a significant increase in SA‐β‐gal activity (Figure S5c). The SA‐β‐gal increase was most prominent in the KDs of *PRPF4* and *SNRPB*. When proliferation was assessed, all KDs also showed a significant decrease in proliferation rate at 8 days of induction. The KD for *PRPF8* was the least effective at impairing proliferation, possibly reflecting KD efficiency (Figure S5d). *p21* and *CCND2* were significantly upregulated in cells induced for the KD of *SNRNP70*, *PRPF8*, *PRPF4*, and *SNRPB* (Figure S5e). *IL1A* was marginally elevated by the depletion of *SNRNP70*, *PRPF8* and *SNRPB*, while *IL1B* was not significantly upregulated. Lastly, *CCND1* was upregulated in response to the KD of *PRPF4* only. Overall, our results indicate that the depletion of each spliceosome component can promote senescence. Differences in the expression of RNA senescence markers may point to distinct mechanisms employed to induce senescence when different components are depleted. Therefore, a cumulative drop in the expression of several spliceosome components may be required to activate the full set of senescence markers.

The shRNA targeting *SF3B1* provoked significant senescence‐associated splicing shifts in the set of eight validated alternative splicing events while splicing was unaffected in *shScramble* cells (Figures 4d and S5f). However, the amplitude of the alterations varied between timepoints. For instance, ΔPSIs for *MYLK* (IR and ES), *CTSA* and *DDX5* peaked at day 5 but were partially restored at day 10. Interestingly, several shifts were observed 48 h post‐induction in *MAGED2, MYLK* (*IR* and *ES*), *MIA2*, *MINK1*, and *MDM4*, which precedes the increase in expression of senescence markers (see Figure 4c). This indicates that SF3B1 may directly control these alternative splicing events. Exclusion of exon 8 in *MDM4* was the most reactive event following *SF3B1* depletion. Notably, this event in *MDM4* was also the most altered both during RSEN in BJ cells and when senescence was induced by splicing inhibitors (Figures 1h and 3d). As for the other components, the KD of *PRPF4* significantly shifted the alternative splicing of *MIA2, MINK1*, and *MDM4* (*p* < 0.05, ΔPSI >|10%|), with *MDM4* being once again, the most reactive unit in the set (Figure S5g). The knockdown of *SNRPB* affected *MINK1*, *MYLK* (*ES*) and DDX5 (opposite shift to RSEN (Figures 1h and 3d)). Lastly, the KD of *SNRNP70*, and *PRPF8* did not affect the alternative splicing of our senescence‐associated events (Figure S5g), possibly reflecting the lower efficiency of the respective shRNAs (Figure S5b).

In summary, the KD of *SF3B1* induces senescence in a manner comparable to Herboxidiene and Pladienolide B. The KD of both *PRPF4* and *SNRPB* also provokes senescence phenotypes and alters many senescence‐associated splicing events. A global defect in the expression of various spliceosome components, such as the one observed during RSEN, is therefore likely to provide a strong impulse towards the senescent state.

### 3.4 MDM4 variants contribute to senescence and improve survival

MDM4 is a p53 inhibitor that operates by binding to the p53 transactivation domain (Marine & Jochemsen, 2005). MDM4 is also required for p53 ubiquitination by MDM2 (Wang et al., 2011). The alternative splicing of *MDM4* can produce several variants (J. Wu et al., 2021) but only two were detected in our study: the main variant termed *MDM4‐FL* (full‐length) and a smaller, truncated variant termed *MDM4‐S* (small) generated by the skipping of exon 6 (68 bp). Due to the insertion of a premature stop codon, MDM4‐S lacks the RING domain essential for dimerization with MDM2 (Tanimura et al., 1999) but still contains the p53‐binding domain (Rallapalli et al., 1999). Consequently, MDM4‐S binds to p53 more efficiently than its full‐length counterpart (Rallapalli et al., 1999, 2003). While the expression level of *MDM4* has recently been associated with senescence (Mejía‐Hernández et al., 2022; Yano et al., 2021), how its splicing variants contribute to senescence remains largely unexplored.

**FIGURE 5:**
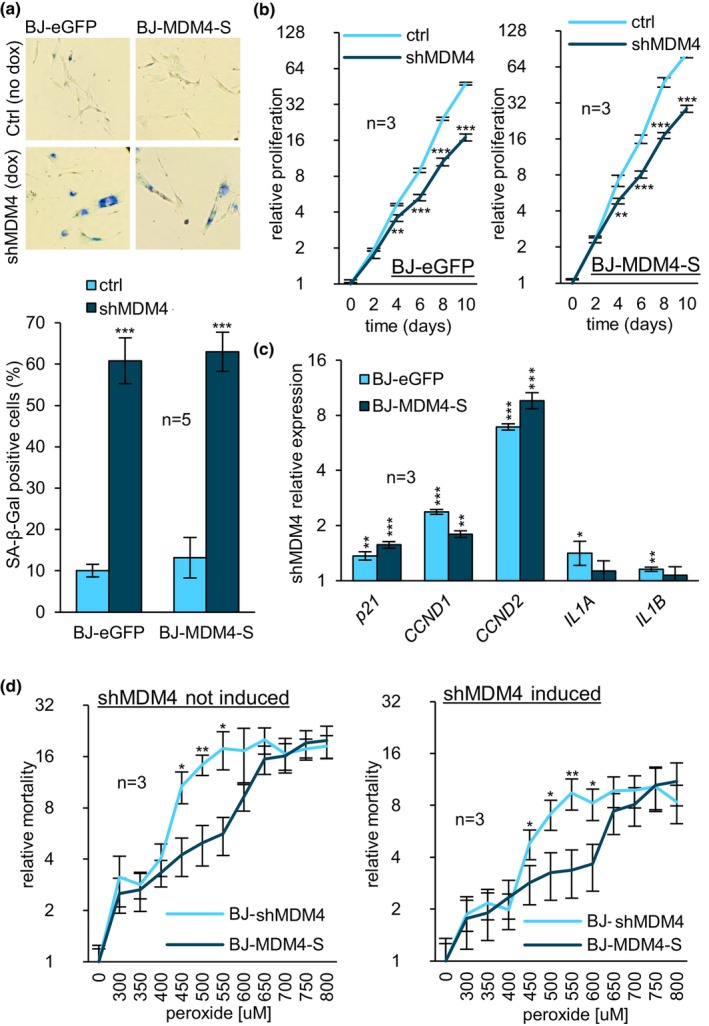
*MDM4* alternative splicing regulates senescence and survival. (a) BJ‐eGFP (left) and BJ‐MDM4‐S (right) cells were stained using the SA‐β‐Gal method. (b) Proliferation rate was measured using the Crystal Violet staining assay. Data from induced cells was statistically compared to non‐induced samples of corresponding timepoints. (c) Relative expression of senescence RNA markers in shRNA‐induced cells was quantified in BJ‐eGFP and BJ‐MDM4‐S cells by qRT‐PCR. Data for each gene was normalized and statistically compared to non‐induced samples. (d) Survival was monitored using the Promega CellTox™ kit in BJ‐shMDM4 and BJ‐MDM4‐S cells treated with the indicated concentration of hydrogen peroxide for 2 h. BJ‐shMDM4 was used as control instead of BJ‐eGFP to avoid eGFP interference with the Promega CellTox™ kit. BJ‐shMDM4 and BJ‐MDM4‐S were either non‐induced (left panel) or induced (right panel) for 24 h following hydrogen peroxide treatment. Data were normalized to non‐treated samples (NT). Mortality values from BJ‐MDM4‐S were statistically compared to those of BJ‐shMDM4 at corresponding hydrogen peroxide concentrations. Doxycycline induction was performed for 10 days. BJ cells were infected for the inducible expression of a shRNA targeting all variants of *MDM4*. BJ‐shMDM4 were infected to express constitutively *eGFP* (BJ‐eGFP) or *MDM4‐S* (BJ‐MDM4‐S). Means were compared using Student's t‐test. *p‐value <0.05, **p‐value <0.01, ***p‐value <0.001.

Throughout our study, the shift in *MDM4* alternative splicing, from *MDM4‐FL* to *MDM4‐S*, was the most reproducible (summarized in Table S2), and its amplitude, the largest in most senescence models (summarized in Figure S6a). To investigate the independent contribution of *MDM4‐S*, a BJ cell line inducible for a global KD of *MDM4* was first generated (BJ‐shMDM4). The depletion was confirmed by qRT‐PCR and the PSI values upon KD were unchanged, indicating that the variants were equally affected by the KD (Figure S7a, “top”). Using these inducible MDM4‐KD cells, we generated two additional cell lines: one constitutively expressing recombinant *MDM4‐S* (BJ‐MDM4‐S), and control cells expressing eGFP (BJ‐eGFP). Since the recombinant *MDM4‐S* does not contain the targeted sequence, its expression is protected from the effect of the shRNA (Figure S7a, “bottom”). After a 10‐day induction, the KD of endogenous *MDM4* transcripts provoked an important increase in SA‐β‐gal activity in both BJ‐MDM4‐S and BJ‐eGFP cells (Figure 5a). While BJ‐eGFP and BJ‐MDM4‐S cells displayed significant decreases in proliferation rate following the *MDM4* KD, the relative difference between both cell lines was marginal (Figure 5a). This result suggests that recombinant *MDM4‐S* does not impair the onset of senescence triggered by the drop of endogenous *MDM4*. Following the KD of endogenous *MDM4*, the expression level of *p21*, *CCND1* and *CCND2* in both BJ‐eGFP and BJ‐MDM4‐S was increased (Figure 5c). Notably, *IL1A* and *IL1B* were remarkably less elevated in BJ‐eGFP and BJ‐MDM4‐S cells compared to RSEN and senescence induced by splicing inhibition (Figures 5c, 1c and 3c). To investigate whether SIPS onset could be affected by *MDM4‐S*, we treated BJ‐eGFP and BJ‐MDM4‐S cells with hydrogen peroxide. While no difference in SA‐β‐gal activity was noted (Figure S7b), slight differences in mortality and confluency were observed during treatment at a higher concentration of hydrogen peroxide (data not shown), suggesting a potential role for *MDM4‐S* in cell survival. Toxicity was therefore monitored in BJ‐eGFP and BJ‐MDM4‐S cells treated with increasing doses of hydrogen peroxide. Results confirmed that *MDM4‐S* expression significantly improved the survival of treated cells, irrespective of the endogenous *MDM4* expression status (Figure 5d). The KD of endogenous *MDM4* also promoted a significant decrease in mortality at a broad range of concentrations, independently of recombinant *MDM4‐S* level (Figure S7c), indicating that the loss of *MDM4‐FL* improves cell survival. Furthermore, the combination of knocking down endogenous *MDM4* and expressing recombinant *MDM4‐S* had an additive impact on cell survival (Figure S7d). These results suggest antagonistic functions for MDM4‐FL and MDM4‐S in cell survival. Next, since MDM4‐S has been described as a negative (Rallapalli et al., 1999, 2003) and positive (Bardot et al., 2015; Dewaele et al., 2015; Pant et al., 2017) regulator of p53, we tested the impact of *MDM4‐S* overexpression on p53 transactivation activity following oxidative stress. Of three p53 transactivation targets responding to hydrogen peroxide (*p21*, *GADD45A*, and *GDF15*), and whose response was lost upon Pifithrin‐α treatment (Figure S7e “left”), none were negatively affected by the expression of *MDM4‐S* (Figure S7e “right”). Notably, as *MDM4‐S* improved cell survival of peroxide‐treated cells (Figure 5d), Pifithrin‐α significantly reduced it (Figure S7f), further indicating that *MDM4‐S* does not inhibit p53.

Since the KD of endogenous *MDM4* provoked senescence independently of *MDM4‐S*, we tested the functional impact of *MDM4‐FL* by using a shRNA targeting the *MDM4‐FL* variant specifically. Despite the small size of the alternative exon 6 (68 nt), we identified one shRNA that elicited an 8.8% drop in *MDM4‐FL* after 10 days of induction (Figure S6b). This mild KD was nevertheless sufficient to increase SA‐β‐gal activity by 5% (Figure S6c). The proliferation rate was also slightly decreased, and the senescence RNA markers *p21*, *CCND1*, and *CCND2* were significantly upregulated (Figure S6d,e). Although the effects were moderate, our results are consistent with the previous conclusion that a specific drop in *MDM4‐FL* level promotes senescence.

Overall, we consistently observed a splicing shift from *MDM4‐FL* to *MDM4‐S* in a variety of senescence models, including senescence induced by splicing defects. Senescence is also promoted by the global KD of endogenous *MDM4* in a *MDM4‐S‐*independent manner, suggesting that the loss of *MDM4‐FL* is the primary contributor to senescence. Lastly, the reduction in *MDM4‐FL* and the increase in *MDM4‐S* individually and collectively enhanced cell survival. Therefore, the convergence of various senescence‐inducing stimuli and spliceosome‐related defects towards the regulation of *MDM4* splicing suggests a role for *MDM4* variants in both sensing and responding to defective splicing activity.

## 4 DISCUSSION

Following excessive intracellular or extracellular stresses, senescence can stop the proliferation of damaged cells, thereby preventing malignant transformation. The phenotypes associated with cellular senescence vary between different cell types and senescence programs (Gil, 2023; Kumari & Jat, 2021). Common senescence phenotypes include the activation of the cyclin‐dependent kinase inhibitor p21‐dependent pathway (Campisi, 2013), accumulation of the lysosomal β‐galactosidase enzyme (Dimri et al., 1995) and the release of proinflammatory factors (SASP) (Coppé et al., 2010; Faget et al., 2019; Freund et al., 2010). In addition, senescent cells often display resistance to apoptosis, leading to improved survival (Hu et al., 2022). While the insults triggering senescence are often linked to DNA damage, the specific pathways leading to the umbrella of associated phenotypes are not fully understood. Our analysis confirms previous studies that associate deficits in the expression of splicing regulators with senescence (Dong et al., 2018; Kwon et al., 2021). The decreased expression of splicing regulators *hnRNP F*, *SRSF3*, and *SRSF7* observed in most of our senescence models, likely contribute to senescence given that their individual depletion induces senescence in different systems (Hong et al., 2023; Kwon et al., 2021; Tang et al., 2013; Xu et al., 2020). *PTBP1* and *FUS* were also found downregulated in RSEN BJ and IMR‐90 cells, in SIPS BJ and HCT116 senescent cells, and strongly associated with early passage quiescent BJ cells. Previous studies have shown that the depletion of PTBP1 may provoke G_0_ and G_1_ arrests (Ji et al., 2018), while the depletion of FUS leads to cell cycle impairments (Ward et al., 2014). However, what triggers these drops in expression and how they contribute to senescence remain to be investigated.

Our RNAseq analysis revealed a general decline in spliceosome components associated with RSEN. We validated some of these deficits in WI‐38 and IMR‐90 cells undergoing RSEN, in senescent BJs induced by telomere uncapping as well as in BJ, WI‐38, and HCT116 cells undergoing SIPS. While *SNRPB*, *PRPF4* and *SF3A2* were the most downregulated in BJ cells undergoing RSEN, drop in *U2AF1* and *SNRPA1* were detected in nearly all our senescence models. Altogether, this supports the view that the associated deficit in spliceosome components is a universal hallmark of senescence. Why this general drop occurs, however, remains to be investigated. One potential mechanism could involve transcription factors such as MYC, which is often downregulated during senescence and positively regulates the expression of several splicing factors (Anczuków & Krainer, 2015; Marthandan et al., 2015; Wu et al., 2007).

Such drops in the expression of constitutive spliceosome components would be expected to reduce splicing activity, therefore promoting intron retention events (AlShareef et al., 2017; Kashyap et al., 2015). Consistent with this view, we observed a significant increase in retained introns during RSEN. Showing that inhibiting splicing with Pladienolide B and Herboxidiene robustly induces senescence independently of DNA damage suggested that the senescence‐associated global reduction in the expression of splicing factors might contribute to senescence. Consistent with this view, the individual shRNA‐mediated depletion of spliceosome components *SF3B1, SNRP70, PRPF8, PRPF4*, and *SNRPB* promoted senescence. Targeting different spliceosome components however revealed differences in the activation of the senescence markers. For instance, the increase in *p21* occurred with the depletion of *PRPF4* but not *SF3B1*, whereas the reverse was seen for *IL1A* and *IL1B*. Thus, the cumulative effect of individual spliceosome‐related defects may help trigger a full senescence program. Notably, SF3B1 is one of the most mutated splicing factors across multiple types of cancer, often leading to extensive splicing aberrations (Seiler et al., 2018; Shiozawa et al., 2018). While the exact contribution of these mutations to carcinogenesis is still unclear, they have been associated with defective telomere maintenance, DNA damage and immune infiltration deregulation (Zhou et al., 2020). Due to frequent mutations in spliceosome components and the often observed alterations in their expression during cancer (Seiler et al., 2018; Urbanski et al., 2018), senescence could be triggered in this context and may serve as a primary defense against oncogenic splicing defects.

Given the senescence‐associated drops in the expression of splicing factors, alterations of alternative splicing are expected. Among the alternative splicing changes that we associated with RSEN BJ cells, many were also occurring in IMR‐90 and WI‐38 cells. Similar changes were detected when senescence was provoked by TIN2DN, hydrogen peroxide, the knockdown of spliceosome components, and splicing inhibitors. The absence of noticeable DNA damage induced by splicing inhibitors suggests that those common splicing shifts are not directly caused by DNA stress but are rather part of a downstream regulatory network. While most splicing changes are consistent across our models, their amplitudes vary in proportion to the induction of senescence markers, suggesting that splicing changes occur only in cells in which the senescence program has been triggered. Considering that these shared occurrences are often in genes that have been associated with senescence and given that they occur prior to the induction of senescence, they may serve as functional nodes where deficiencies in splicing factors converge to mediate senescence. Among these, the retention of *PSMC3IP* first intron was consistently enhanced in RSEN and SIPS, and when senescence was induced by splicing inhibitors. This event has previously been suggested to produce a dominant‐negative variant (Peng et al., 2013) which, given that repressing PSMC3IP inhibits cell proliferation (Ding et al., 2019), could contribute to senescence‐associated proliferation arrest.

Finally, the alternative splicing event whose shift was most consistent and often the strongest among our diverse models was the 68‐nt exon skipping event in *MDM4*. Previous studies have associated *MDM4* with senescence‐like features. For instance, overexpression of *MDM4‐FL* in TIG‐3 cells affected cell proliferation and p21 expression (Yano et al., 2021). Also, while the simultaneous knockdown of all *MDM4* transcripts induces cell proliferation arrest and SA‐β‐gal activity in a prostate cancer cell line (Mejía‐Hernández et al., 2022), the specific contributions of *MDM4‐FL* and *MDM4‐S* were not addressed. Here, we found that a specific drop of the *MDM4‐FL* variant is responsible for this effect. Overexpressing *MDM4‐S* did not affect the onset of senescence but it improved cell survival, which is an important feature of senescence (Soto‐Gamez et al., 2019). The decrease expression of *MDM4‐FL* also improved cell survival independently of *MDM4‐S* level, highlighting the antagonistic functions of *MDM4‐FL* and *MDM4‐S*. While p53 was not essential for splicing defects to elicit senescence, it is still possible that MDM4‐S could regulate the survival of senescent cells via p53 (Bardot et al., 2015; Dewaele et al., 2015; Pant et al., 2017; Rallapalli et al., 1999, 2003). While our data indicate that *MDM4‐S* does not inhibit p53, a slight increase in *GADD45A* (Figure S7e) in cells overexpressing *MDM4‐S* raises the possibility that p53 is positively regulated by *MDM4‐S*. However, given that *GADD45A* is the only regulated target from the set, *MDM4‐S* may also act independently from p53 (Klein et al., 2021).

The molecular mechanisms through which SF3B1 and other splicing factors modulate *MDM4* exon 4 inclusion remain unaddressed. Future studies will be crucial to clarify how distinct splicing factor deficiencies converge on this specific splicing event. Given that SF3B1 is a core component of the U2 snRNP that recognizes the branch site of all U2‐dependent introns, its interaction with the branch site associated with *MDM4* exon 4 may be more sensitive to the level and activity of SF3B1. It would also be revealing to investigate how reductions in *PRPF4* and *SNRPB* expression elicit a similar effect on *MDM4* splicing, and whether their impact is functionally linked to SF3B1 or represents independent mechanisms of splicing regulation. Such investigations could provide valuable insights into the complex interplay between splicing regulatory proteins.

In summary, our work indicates that a deficit in the expression of splicing factors and its associated splicing inhibition is not only a hallmark but a functional aspect of senescence. By reducing splicing activity, senescent cells may promote alternative splicing events that elicit or further reinforce the senescence state. Given the role of senescent cell accumulation in physiological aging (Baker et al., 2008, 2011, 2016) and that splicing defects, including increased intron retention, are also established phenotypes of aging (Bhadra et al., 2020; Deschênes & Chabot, 2017), it is possible that defective splicing contributes to aging in part through senescence. Among these alternative splicing events, the shift leading to a drop of *MDM4‐FL* in favor of *MDM4‐S* was ubiquitous. The decrease in *MDM4‐FL* and the consequent increase in *MDM4‐S* contribute to both senescence onset and cell survival. While the molecular mechanism by which MDM4‐S plays its part remains to be addressed, its documented ability to inhibit p53 may help prevent programmed cell death in senescent cells. The *MDM4* splicing shift has been previously noted in the response to the KD of *PRMT5* (Bezzi et al., 2013), *SRSF10* (Sohail et al., 2021), *SF3A3* (Chen et al., 2022) and *SRSF3* (Dewaele et al., 2015), treatment with spliceostatin (Bezzi et al., 2013) and treatment with various inhibitors of the SR protein kinase CLK1 (Bezzi et al., 2013; Sohail et al., 2021). Here, we showed that *MDM4* splicing is highly sensitive to senescence‐inducing defects such as the KD of *SF3B1*, *PRPF4*, *SNRPB*, and treatments with Pladienolide B and Herboxidiene. Overall, *MDM4* remarkable responsiveness to spliceosome‐related aberrations makes it ideally suited to act as a splicing defect sensor, as already proposed by Bezzi et al. (Bezzi et al., 2013). Thus, we have demonstrated that *MDM4* splicing not only responds to spliceosome‐related defects but also participates to the onset of senescence, thereby providing crucial defense against the dysregulation of homeostasis.

## AUTHOR CONTRIBUTIONS

MDe, FR, and BC contributed to the study design and conceptualization. MDe, MDu, MAO and APV acquired, analyzed, and interpreted the data. MDe and MAO prepared figures. MDe and BC drafted the manuscript, which was revised by MDu, MAO, APV and FR. BC supervised the study.

## FUNDING INFORMATION

This work was supported by a grant from the Canadian Institutes of Health Research [PJT‐165966] to BC. M. Deschênes is currently supported by a scholarship from the Fonds de Recherche du Québec – Santé (FRQS).

## CONFLICT OF INTEREST STATEMENT

The authors declare no competing interests.

## Supporting information


Figure S1.
Figure S2.
Figure S3.
Figure S4.
Figure S5.
Figure S6.
Figure S7.

Table S1.

Table S2.

Table S3.

Table S4.

## Data Availability

Raw RNAseq data and data processed for gene expression and alternative splicing analysis were deposited at the Gene Expression Omnibus (GEO) repository. Accession number: GSE245356. TOKEN FOR REVIEWERS: ypklowauzvkbter.
