# Supplementary figures and images for: A defective splicing machinery promotes senescence through MDM4 alternative splicing
Source: Aging Cell. 2024 Aug 8;23(11):e14301. doi: 10.1111/acel.14301 (PMC11561654; doi:10.1111/acel.14301)

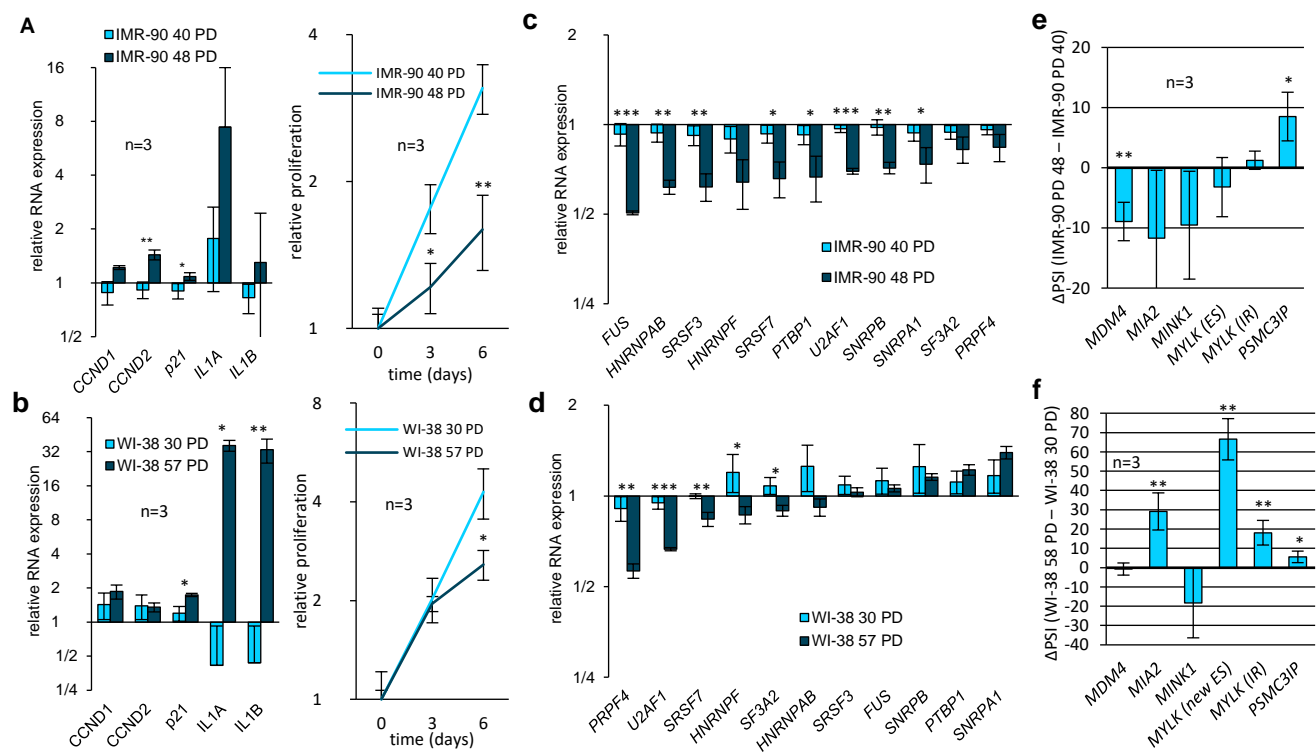

**Figure S1**

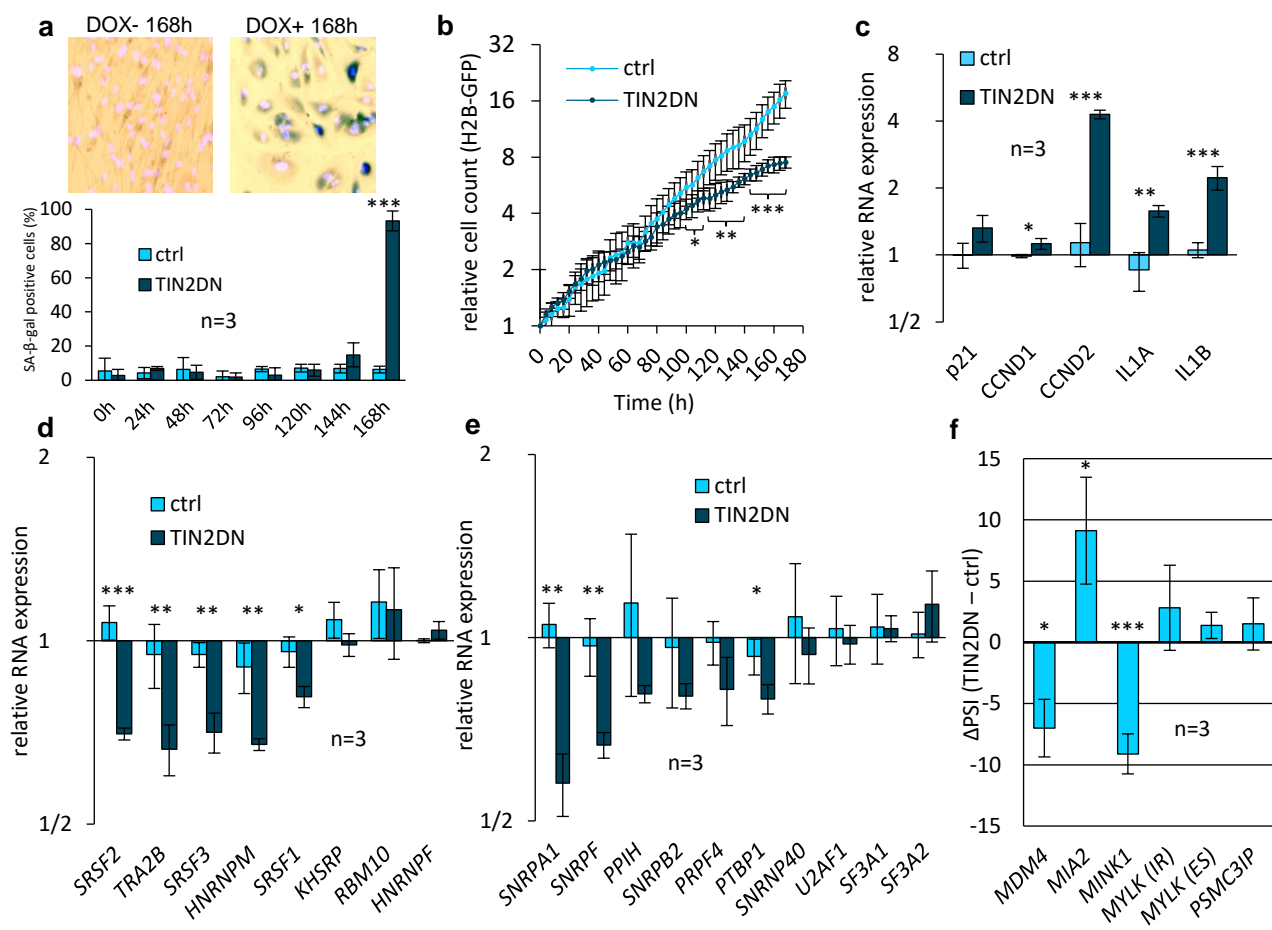

**Figure S2**

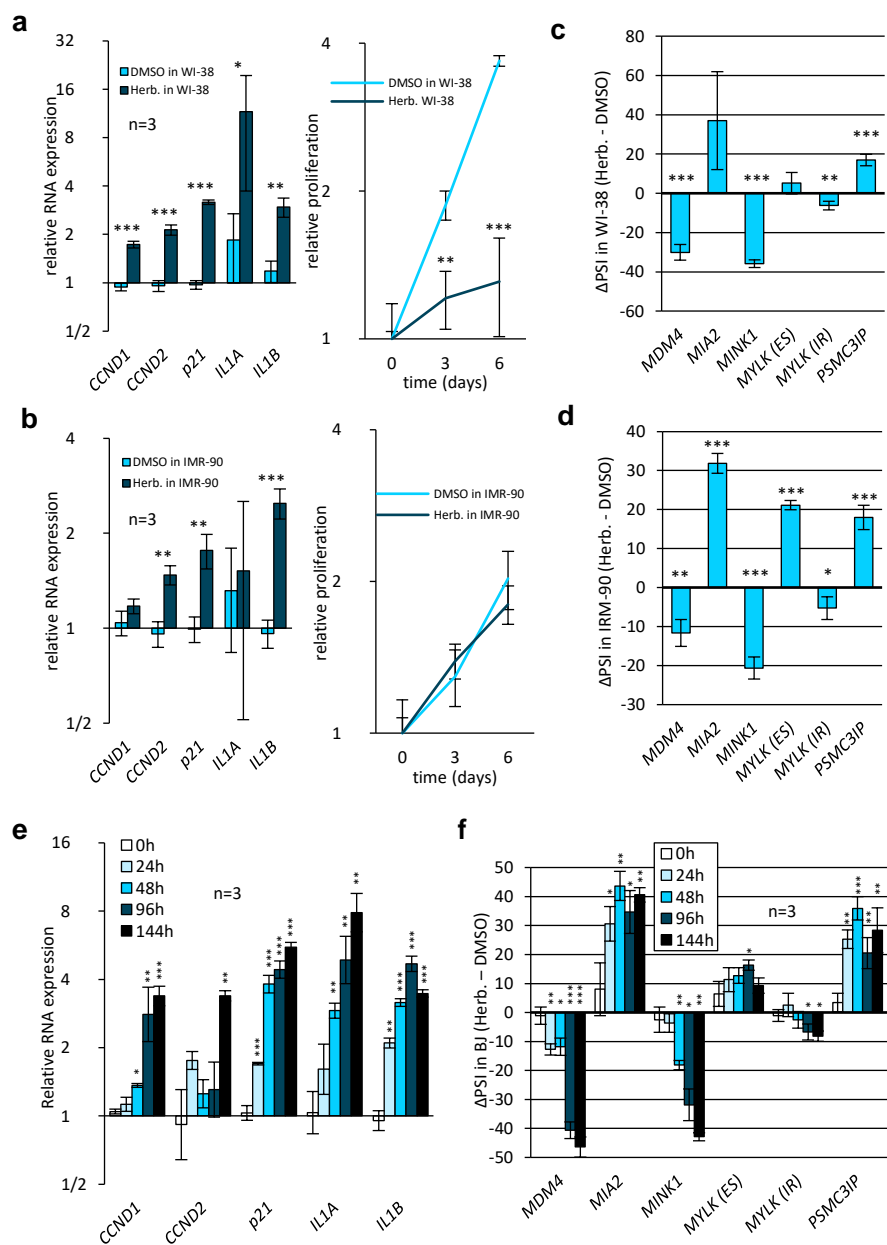

**Figure S3**

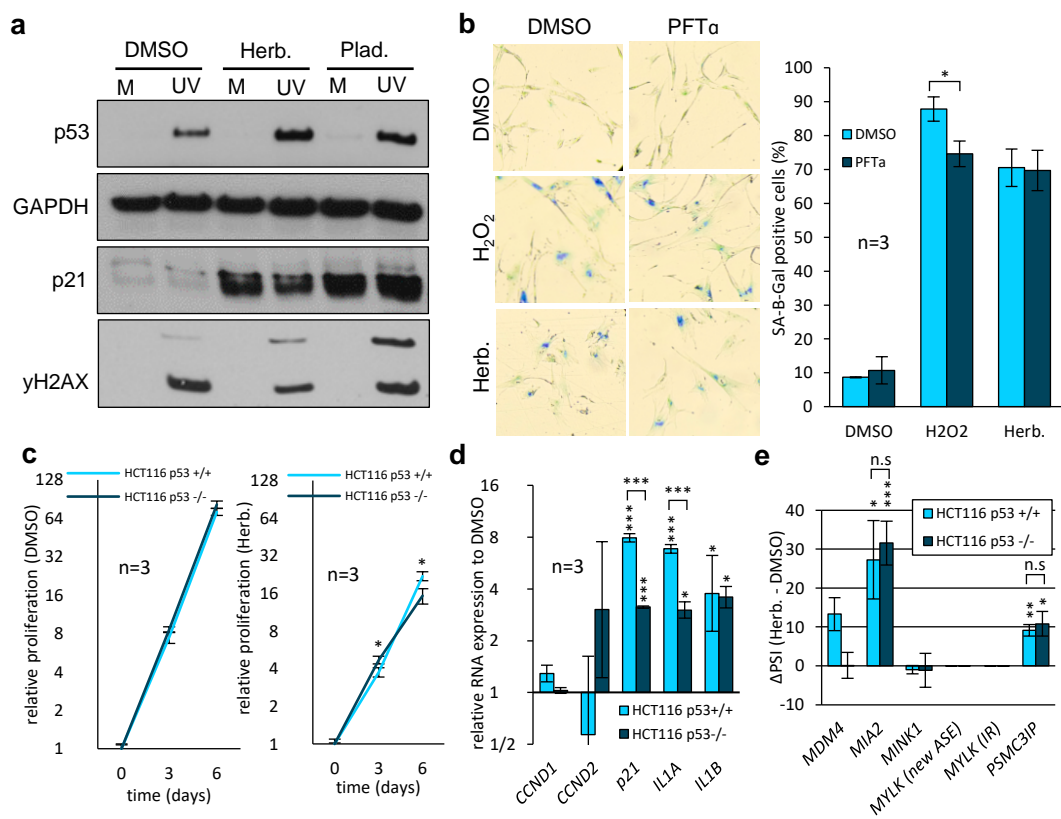

**Figure S4**

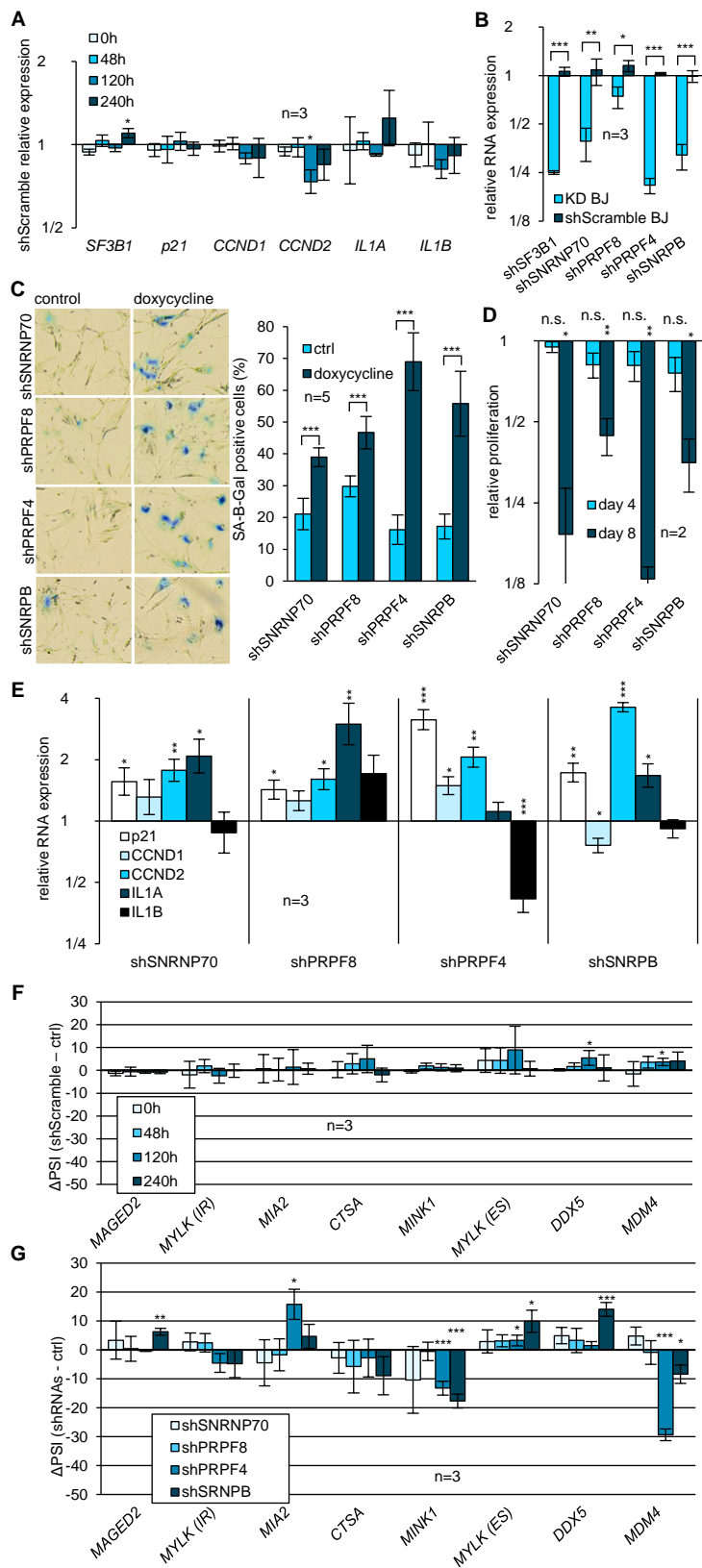

Figure S5

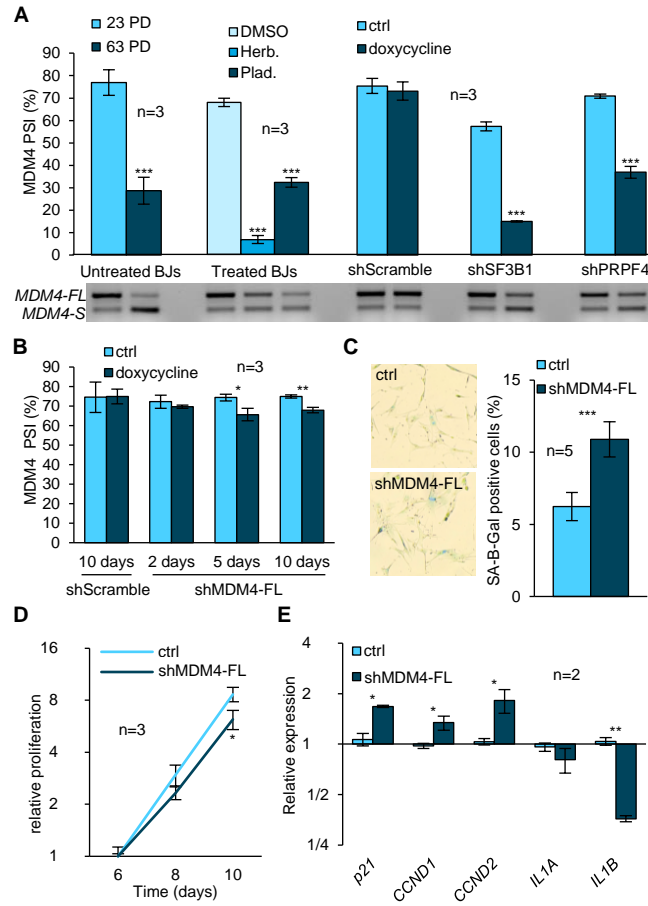

**Figure S6**

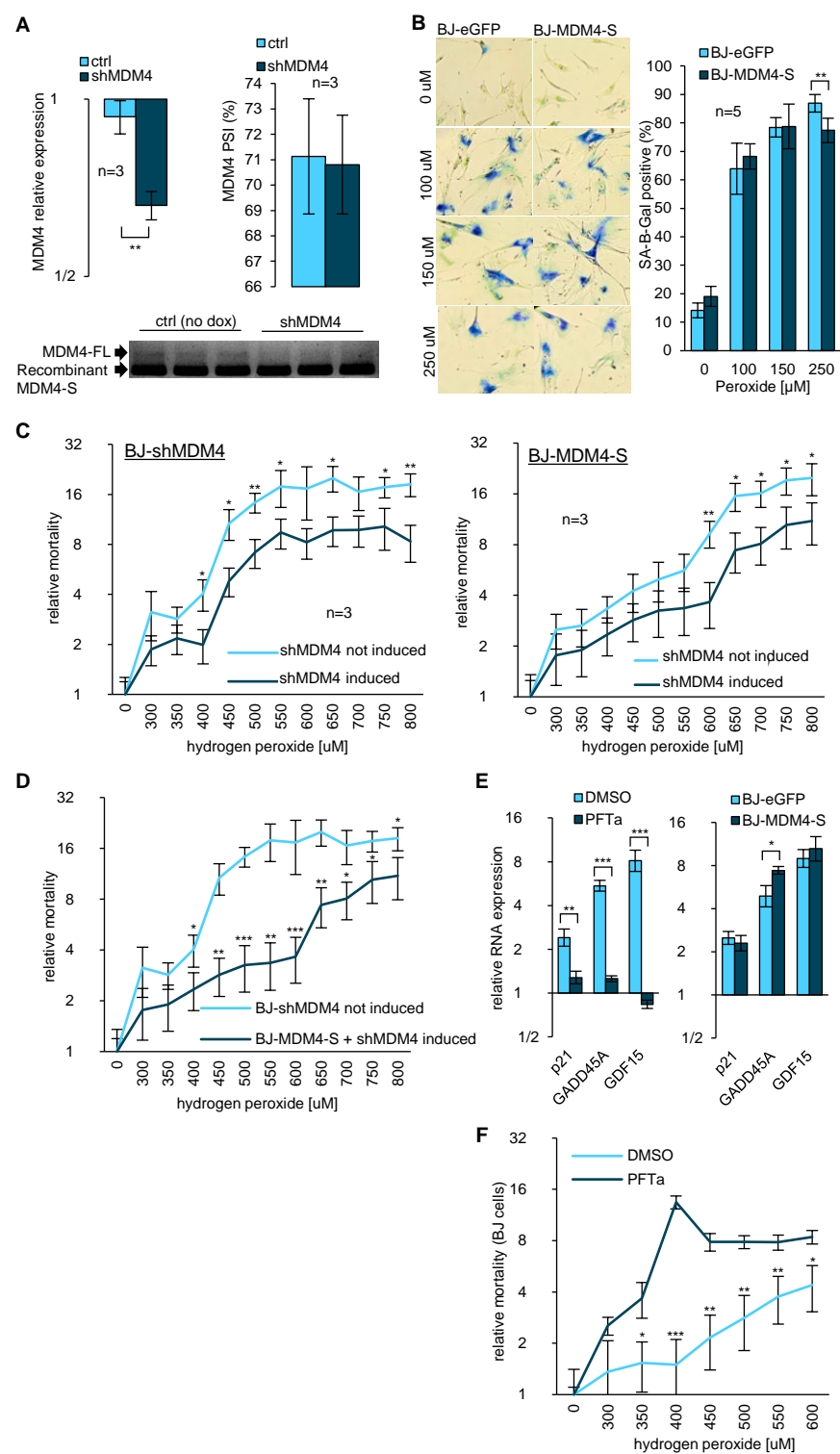

**Figure S7**

Supplement: Supplementary file 1 — Figure S1. Figure S2. Figure S3. Figure S4. Figure S5. Figure S6. Figure S7. [file ACEL-23-e14301-s004.pdf]
