# Supplementary material for: A defective splicing machinery promotes senescence through MDM4 alternative splicing
Source: Aging Cell. 2024 Aug 8;23(11):e14301. doi: 10.1111/acel.14301 (PMC11561654; doi:10.1111/acel.14301)
Supplement: Supplementary file 2 — Table S1. [file ACEL-23-e14301-s001.pdf]

Table S1 Senescent BJ cells preferentially downregulate splicing factors

splicing regulators	BJ_senescent vs BJ_young Log2-ratio	BJ_senescent vs BJ_young Ratio	BJ_senescent Avg	BJ_senescent Stdev	BJ_young Avg	BJ_young Stdev
HIST1H4H	3.6	12.2	13.8	0.8	1.1	0.1
SYT1	2.7	6.3	6.2	0.1	1.0	0.1
HIST2H4A	2.4	5.3	26.1	1.8	4.9	0.2
HIST1H2BK	1.9	3.6	69.3	5.5	19.1	0.4
PIK3R3	1.8	3.4	7.1	0.1	2.1	0.2
CTSD	1.2	2.3	287.8	1.4	123.7	1.4
TANC1	1.2	2.3	22.2	0.5	9.7	0.4
MSI2	1.0	2.0	34.7	0.9	17.5	1.0
AEBP1	0.9	1.8	387.8	2.6	213.7	1.8
DHX40	0.8	1.8	45.7	1.6	25.4	0.9
RNF213	0.8	1.7	65.3	3.3	37.4	2.3
BHLHE40	0.8	1.7	14.2	0.4	8.3	0.2
CWC25	0.8	1.7	14.8	2.8	8.7	0.5
RNPC3	0.8	1.7	13.1	1.2	7.8	0.2
SLFN5	0.8	1.7	65.8	2.4	39.0	0.3
CUX1	0.7	1.6	43.6	0.5	26.5	0.3
CCDC47	0.7	1.6	109.7	1.4	69.5	0.1
DDX42	0.7	1.6	138.0	1.8	87.6	0.8
JUP	0.6	1.6	16.3	0.5	10.5	0.3
LUC7L3	0.6	1.5	174.5	2.5	113.9	2.7
AHNAK	0.6	1.5	554.1	11.7	373.1	2.3
DHX8	0.6	1.5	42.4	0.1	28.6	0.1
DDX5	0.6	1.5	810.4	5.2	550.5	20.4
FAM50B	0.6	1.5	17.7	0.3	12.1	0.1
CLTC	0.5	1.5	388.6	10.0	268.0	7.9
MED1	0.5	1.4	43.2	0.9	30.0	0.5
NEDD1	0.5	1.4	98.0	0.3	70.2	3.7
BCL2L2	0.5	1.4	59.8	0.8	42.9	0.0
MYPN	0.5	1.4	32.3	0.9	23.2	0.6
COIL	0.5	1.4	17.5	0.0	12.6	0.1
CDK12	0.5	1.4	36.7	1.0	26.5	0.2
SMARCE1	0.5	1.4	179.9	2.0	130.7	0.0
RANGAP1	-0.5	0.7	128.6	3.2	175.8	2.2
GPKOW	-0.5	0.7	18.7	0.0	25.6	0.3
RAVER1	-0.5	0.7	25.4	0.1	34.8	0.8
TPM3	-0.5	0.7	670.3	28.2	920.4	38.1
RBM14	-0.5	0.7	28.2	0.5	38.7	0.7
EEF1D	-0.5	0.7	176.6	1.9	243.4	2.9
SMC4	-0.5	0.7	104.8	2.6	144.7	0.2
PFKP	-0.5	0.7	94.1	0.8	130.0	1.1

**TRIM28**	-0.5	0.7	150.3	2.0	207.8	6.6
**KIF23**	-0.5	0.7	55.3	0.8	76.5	1.3
**SMARCC1**	-0.5	0.7	33.2	0.2	46.0	0.6
**SRRT**	-0.5	0.7	57.8	0.6	80.3	0.4
**RSRC1**	-0.5	0.7	26.7	1.5	37.1	1.0
**MOV10**	-0.5	0.7	28.8	0.2	40.0	2.1
**NCAPG2**	-0.5	0.7	33.4	0.8	46.5	0.8
**HCFC1**	-0.5	0.7	16.3	0.4	22.6	0.9
**PRDX1**	-0.5	0.7	557.3	8.0	775.6	4.6
**SET**	-0.5	0.7	273.2	0.1	381.4	8.6
**PSMC3**	-0.5	0.7	320.2	33.4	447.8	41.9
**POLR2E**	-0.5	0.7	183.7	7.3	256.9	6.0
**PDIA3**	-0.5	0.7	500.7	2.4	700.9	5.8
**HNRNPA3**	-0.5	0.7	296.5	11.5	415.2	10.8
**MSH6**	-0.5	0.7	35.6	0.1	49.9	0.9
**ALDOA**	-0.5	0.7	684.2	4.6	959.3	28.7
**FUS**	-0.5	0.7	342.4	7.0	480.4	14.1
**NOLC1**	-0.5	0.7	40.4	0.7	56.7	0.3
**PCBP1**	-0.5	0.7	184.9	1.3	260.5	4.4
**KARS**	-0.5	0.7	90.8	0.3	127.9	1.2
**RARS**	-0.5	0.7	104.6	2.3	147.4	8.1
**EXOSC2**	-0.5	0.7	21.1	1.5	29.8	1.1
**VCL**	-0.5	0.7	165.8	2.8	234.1	4.3
**NUP107**	-0.5	0.7	36.0	0.9	50.9	0.5
**CCT7**	-0.5	0.7	330.7	25.9	467.4	46.6
**SMC3**	-0.5	0.7	33.7	0.2	47.7	0.7
**RFC3**	-0.5	0.7	14.5	0.5	20.5	0.4
**KHSRP**	-0.5	0.7	160.5	1.0	227.1	1.4
**GNL3**	-0.5	0.7	69.4	0.0	98.3	2.0
**PPP4C**	-0.5	0.7	117.5	5.7	166.6	9.1
**TARS**	-0.5	0.7	125.4	2.8	178.0	10.8
**MCM6**	-0.5	0.7	27.0	0.5	38.4	0.9
**DDX1**	-0.5	0.7	93.5	1.2	133.3	2.6
**SHCBP1**	-0.5	0.7	33.3	1.6	47.5	0.5
**CCT2**	-0.5	0.7	145.8	1.7	208.4	4.1
**TCERG1**	-0.5	0.7	37.2	1.2	53.3	0.8
**DNMT1**	-0.5	0.7	53.2	1.9	76.4	2.8
**RFC5**	-0.5	0.7	13.5	0.3	19.4	0.8
**PRPF38A**	-0.5	0.7	18.1	0.5	26.0	0.5
**ILF3**	-0.5	0.7	120.1	2.5	172.7	1.2
**LSM7**	-0.5	0.7	178.6	7.5	256.9	21.3
**NCAPH**	-0.5	0.7	16.9	0.1	24.4	0.1
**KIF11**	-0.5	0.7	23.2	0.3	33.5	1.0
**PSMA7**	-0.5	0.7	216.6	0.0	313.1	1.7
**SERBP1**	-0.5	0.7	230.1	2.2	333.5	2.1
**COPG1**	-0.5	0.7	168.7	2.8	244.8	0.7
**ACTL6A**	-0.5	0.7	62.4	5.1	90.6	3.2

**SMARCD3**	-0.5	0.7	23.9	1.4	34.8	0.5
**PTBP1**	-0.5	0.7	169.3	1.8	247.3	1.7
**C1QBP**	-0.5	0.7	125.1	0.8	182.8	4.9
**RIN1**	-0.6	0.7	14.4	0.1	21.1	0.2
**SNRNP40**	-0.6	0.7	30.7	0.9	45.2	1.2
**SCAF8**	-0.6	0.7	13.6	0.4	20.1	0.5
**RPSA**	-0.6	0.7	1071.2	3.9	1581.1	30.3
**SRSF3**	-0.6	0.7	386.1	14.6	572.3	17.8
**SND1**	-0.6	0.7	120.3	2.6	178.3	2.0
**LGALS3**	-0.6	0.7	75.1	0.7	111.4	0.6
**NCAPD3**	-0.6	0.7	16.8	0.3	25.0	0.1
**NUDT21**	-0.6	0.7	79.7	2.1	118.8	0.3
**HNRNPM**	-0.6	0.7	229.0	7.8	342.0	10.6
**TUBB**	-0.6	0.7	864.4	1.1	1294.1	31.9
**HNRNPF**	-0.6	0.7	123.9	3.1	185.7	0.9
**TUBA4A**	-0.6	0.7	20.2	0.3	30.4	0.9
**SRSF7**	-0.6	0.7	114.6	3.6	172.4	0.8
**GAPDH**	-0.6	0.7	3160.5	79.4	4758.1	192.5
**NOP56**	-0.6	0.7	87.6	3.5	132.2	0.5
**CAD**	-0.6	0.7	17.2	0.5	26.0	0.2
**LMNA**	-0.6	0.7	706.7	19.6	1070.0	18.1
**ENO1**	-0.6	0.7	930.0	9.3	1409.3	14.4
**EIF4A1**	-0.6	0.7	712.2	5.9	1080.5	13.1
**DLGAP5**	-0.6	0.7	34.2	0.4	52.1	0.1
**RPS2**	-0.6	0.7	1868.8	42.9	2861.6	81.5
**BAZ1A**	-0.6	0.6	18.2	0.0	28.2	0.3
**PRMT1**	-0.6	0.6	121.4	0.8	188.4	1.6
**GLE1**	-0.6	0.6	14.2	0.4	22.1	0.4
**DKC1**	-0.6	0.6	36.1	0.5	56.2	0.5
**PARP1**	-0.6	0.6	54.2	1.5	84.5	2.4
**HSPB1**	-0.6	0.6	658.8	6.9	1026.9	9.1
**THOC6**	-0.6	0.6	9.2	0.4	14.3	0.4
**PPP1CA**	-0.6	0.6	97.3	0.2	152.2	1.7
**HNRNPAB**	-0.6	0.6	161.0	6.7	252.7	7.5
**CCDC9**	-0.7	0.6	24.0	1.3	38.1	1.6
**HSPA8**	-0.7	0.6	1028.2	10.2	1645.7	60.0
**PAICS**	-0.7	0.6	86.6	0.9	139.5	3.0
**DDX39A**	-0.7	0.6	49.0	1.6	79.1	0.3
**PPIB**	-0.7	0.6	651.2	1.2	1053.2	4.8
**HNRNPD**	-0.7	0.6	304.8	9.7	494.2	22.7
**TRA2B**	-0.7	0.6	89.0	0.2	144.7	1.3
**MSH2**	-0.7	0.6	17.1	0.1	27.8	0.7
**HSPA2**	-0.7	0.6	24.6	0.5	40.0	1.1
**SSRP1**	-0.7	0.6	88.0	1.7	144.5	1.9
**BAG2**	-0.7	0.6	18.1	0.4	29.7	0.9
**PRIM2**	-0.7	0.6	18.9	0.0	31.2	0.6
**NCAPH2**	-0.7	0.6	25.0	1.0	41.3	0.4

**MED8**	-0.7	0.6	28.8	0.7	47.6	0.3
**EXOSC9**	-0.7	0.6	38.3	0.5	63.4	0.8
**ANP32A**	-0.7	0.6	110.0	4.3	184.0	7.3
**FBL**	-0.7	0.6	92.4	8.1	155.4	15.1
**TUBA1B**	-0.8	0.6	1400.2	14.0	2374.9	75.3
**VASP**	-0.8	0.6	99.4	2.8	168.7	10.1
**RFC4**	-0.8	0.6	20.0	0.9	34.2	0.2
**PDIA6**	-0.8	0.6	259.3	0.1	444.0	6.8
**ACTB**	-0.8	0.6	4680.3	82.7	8032.3	73.5
**EXOSC8**	-0.8	0.6	26.8	0.4	46.2	0.6
**EBNA1BP2**	-0.8	0.6	90.0	0.7	156.5	3.3
**SELENBP1**	-0.8	0.6	16.3	0.1	28.6	0.2
**SEC13**	-0.8	0.6	131.5	1.1	231.6	1.9
**MCM5**	-0.8	0.6	43.3	0.4	76.3	3.8
**HSP90B1**	-0.8	0.6	662.3	8.5	1179.5	7.9
**TALDO1**	-0.8	0.6	138.0	0.8	246.3	0.4
**MCM2**	-0.8	0.6	20.1	2.5	36.0	1.1
**FLNA**	-0.9	0.6	674.7	12.0	1223.1	31.9
**RBM3**	-0.9	0.6	241.0	7.3	437.1	13.3
**FASN**	-0.9	0.5	23.8	0.1	43.3	0.9
**HMGB2**	-0.9	0.5	118.2	5.6	215.6	0.2
**VARS**	-0.9	0.5	8.6	0.1	15.8	0.0
**MAGOHB**	-0.9	0.5	30.7	0.5	56.7	0.9
**PDIA4**	-0.9	0.5	119.5	0.4	221.6	0.9
**EEF1E1**	-0.9	0.5	37.8	1.3	70.4	1.1
**IMPDH1**	-0.9	0.5	45.1	0.0	84.5	2.0
**ATAD3A**	-0.9	0.5	27.7	0.5	51.8	0.6
**MIOS**	-1.0	0.5	11.6	0.6	22.8	1.4
**PGD**	-1.0	0.5	87.3	3.1	172.3	0.6
**LMNB1**	-1.0	0.5	32.1	1.3	64.6	1.2
**HSPA5**	-1.1	0.5	328.2	6.6	683.3	14.4
**DSP**	-1.2	0.4	26.2	0.4	58.4	0.4
**ATAD3B**	-1.2	0.4	12.4	0.3	28.1	0.1
**TFRC**	-1.2	0.4	47.0	1.2	110.2	0.1
**TKT**	-1.3	0.4	241.2	1.7	599.6	21.0
**MAP1B**	-1.5	0.3	59.6	0.4	173.7	2.0
**FABP5**	-1.8	0.3	20.3	0.4	69.3	1.9
**S100A10**	-2.0	0.2	57.9	0.2	235.5	6.9
**ACTA2**	-2.3	0.2	124.7	3.9	635.5	4.7
**IGF2BP3**	-4.0	0.1	1.1	0.0	17.2	0.8
