# Supplementary material for: A defective splicing machinery promotes senescence through MDM4 alternative splicing
Source: Aging Cell. 2024 Aug 8;23(11):e14301. doi: 10.1111/acel.14301 (PMC11561654; doi:10.1111/acel.14301)
Supplement: Supplementary file 3 — Table S2. [file ACEL-23-e14301-s005.pdf]

Table S2 Summary of alternative splicing events of interest in various types of senescence

ASEs	REPLICATIVE			TIN2DN	PEROXIDE			SPLICING INHIBITORS					BJ SPLICEOSOME KD					SCORE
	BJ	IMR90	WI38	BJ	BJ	WI38	HCT116	BJ	HIEC	IMR	WI38	HCT116	SNRNP70	SF3B1	SNRPB	PRPF4	PRPF8	
MDM4	-1	-1	0	-1	-1	-1	-1	-1	-1	-1	-1	0	0	-1	0	-1	0	12
MINK1	-1	0	0	-1	-1	-1	-1	-1	-1	-1	-1	0	0	-1	-1	-1	0	12
PSMC3IP	1	1	1	0	1	1	0	1	1	1	1	1	0				0	10
MIA2	1	0	1	1	0	1	0	1	1	1	0	1	0	0	0	1	0	9
MYLK ES	1	0	1	0	0	0	0	1	1	1	0	0	0	1	0	0	0	6
MYLK IR	1	0	1	0	-1	-1	0	-1	0	-1	-1	0	0	-1	1	1	0	2

Legend		
	-1	negative ΔPSI (p-value < 0.05)
	1	positive ΔPSI (p-value < 0.05)
	0	no significant change
	SCORE	absolute value of the sum
