# Supplementary material for: A defective splicing machinery promotes senescence through MDM4 alternative splicing
Source: Aging Cell. 2024 Aug 8;23(11):e14301. doi: 10.1111/acel.14301 (PMC11561654; doi:10.1111/acel.14301)
Supplement: Supplementary file 4 — Table S3. [file ACEL-23-e14301-s003.pdf]

Table S3 small-hairpin RNA (shRNA) sequences

name	target gene	shRNA sequence 5' - 3' (sense)
sh_MDM4L_5+	MDM4 (specific isoform)	GCACAGGATCACAGTATGG
shMDM4_35+	MDM4	GCTCCTGTCGTTAGACCTAAA
sh_scrambled+	N/A	GCGACTAACGCCGAATAGAGA
sh_PRPF4_1+	PRPF4	GCACAAGTCTCTCCGGTCTTT
sh_PRPF8_2+	PRPF8	GCCTCATTCATCGTGCAGTAT
sh_SF3B1_1+	SF3B1	CCTCGATTCTACAGGTTATTA
sh_SNRP70_2+	SNRNP70	GAGACATGCACTCCGCTTACA
sh_SNRPB_2+	SNRPB	AGCCAAAGAACTCCAAACAAG
