# Supplementary material for: A defective splicing machinery promotes senescence through MDM4 alternative splicing
Source: Aging Cell. 2024 Aug 8;23(11):e14301. doi: 10.1111/acel.14301 (PMC11561654; doi:10.1111/acel.14301)
Supplement: Supplementary file 5 — Table S4. [file ACEL-23-e14301-s002.pdf]

Table S4 primer sequences

PRIMERS FOR ALTERNATIVE SPLICING EVENTS (RT-PCR / ddPCR)		
**GENE ID**	**FORWARD PRIMERS**	**REVERSE PRIMERS**
**ABI3BP L (ddPCR)**	CCTCAAACTAAACTGGCTCCCAA	GGTGTATGACACTTCTGGACTTGG
**ABI3BP S (ddPCR)**	CACACCAAACCTGCTCCCAA	GGTGTATGACACTTCTGGACTTGG
**ACTG1**	CCGGGAACAAAAGGCGGG	ATGGAAGGAAACACGGCTCGG
**CBWD3**	GTATCATAACTATTGTGGATTCAAAATATGGATT	TTCATTGATAAGGCCATCAGGTTT
**CTSA**	AGCAGAGGTGAGCTGGCA	CAGTAGTGGAGGTGCTTGGAG
**DDX5 L (ddPCR)**	CTGGCTGTTGTGGTGTGCAAAA	TGTGCCTGTTTTGGTACTGCGA
**DDX5 S (ddPCR)**	GGCCTCCAGAGGGCTAGATGT	TGTGCCTGTTTTGGTACTGCGA
**MAGED2**	CTGAGAACGGAGTCGTTGGGG	TGGAAGCGAGTTAGACCTGCAC
**MAN2C1**	GGAGCGGGTGGAGAAGTTCG	GCGTCAGGAAGCTGGAGAGC
**MDM4**	TCTTGTCACTTTAGCCACTGCT	TCTGAGGTAGGCAGTGTGGG
**MIA2**	CAGCTCGGAATGCTGAAAGAAACC	GAGGAAACATCATCCTACGGTCC
**MINK1**	GAGGACCTCATCTATCGCCACTG	GTCGGGCTTGGCTTTATTCCC
**MYLK (ES)**	GCTCTTATGACCTACAGTCAAACAAG	CCACCGTCTTCTCTGTTGTTTGT
**MYLK (IR)**	GCCCCATTCCATTCTCTGAACA	AGTTTTTCTGCATTGAGCGGG
**NFYA**	CAAGTGGCACAGACTGCTGAAG	GTGTTGGTATTGGCTCCTGTTTGA
**PSMC3IP**	GCGGGAAAGGCGATGAGTAAAG	CGTGTTCCCGCTGTAGGTTC
**RANGRF**	CAAGAAGCCTGGGTCCTCTCTG	TGCAGGTGACAGATTTTCGGGG
**TBRG1 L (ddPCR)**	TCCTTCCCACAGTTCCAGTTTG	TGCATGTTTTCTTCAGAACTTCCAG
**TBRG1 S (ddPCR)**	TCCTTCCCACAGTTCCAGTTTG	AAGAGGCTGCTGCTTCCAG
**TNC L (ddPCR)**	CTGACCCTGCTCTGGAAGACAC	GATGGAGACTGTATAAGGCGTAGCA
**TNC S (ddPCR)**	CTGACCCTGCTCTGGAAGACAC	GAGCCCATGGCAGTGGA
**TPP1 L (ddPCR)**	CTTGAAACGATGCAGACAGGCAG	GTAGGCGTGTAATCTTCCAGCT
**TPP1 S (ddPCR)**	GCTTTCTCAACCCAAGGCTCTAC	GTAGGCGTGTAATCTTCCAGCT
**TTLL11**	CGCTTGCCTTGTGACATCTACTG	GCTCCTGATATGGCTCGGTACAAA
**VCAN L (ddPCR)**	TCAGAGAAAATAAGACAGGTCGAAT	CATCTTCTTCTGCTTCTCCAGAGC
**VCAN S (ddPCR)**	TGCCTACTGCTTTAAACGTCGAAT	CATCTTCTTCTGCTTCTCCAGAGC
**PRIMERS FOR GENE EXPRESSION (qRT-PCR)**		
**GENE ID**	**FORWARD PRIMERS**	**REVERSE PRIMERS**
**CCND1**	CCTCACACGCTTCCTCTCCAGA	CTCCTCCTCTTCCTCCTCCTCG
**CCND2**	TCAAGTGCGTGCAGAAGGACAT	CTTCGCACTTCTGTTCCTCACA
**CDKN1A**	TTGTGCCTCGCTCAGGGGAG	GCGGATTAGGGCTTCCTCTTGG
**CDKN2A (p14)**	CCTCGTGCTGATGCTACTGAGG	CTCCGCCACTCGGGCGCT
**CDKN2A.(p16)**	CTGCCCAACGCACCGAATAGTT	GGTCGGGTGAGAGTGGCGGG
**CDKN2B**	TGAGGACAAAGTGGAGGAGGGC	GTGGAGTTGGGCGAGGGTCT
**CFH**	ACTGGCTGGATACCTGCTCCG	AGCTACTGGAAAGTATGGTCTACGCA
**FUS**	GTGGCTATGAACCCAGAGGTCG	CACCAAATTTATTGAAGCCACCACGG
**HIST1H2AC**	CGCGCCAAAGCGAAATCCCG	GCGGTCAGGTACTCTAACACCG
**HNRNPAB**	AGAAGAAGAACCCGTGAAGAAGGTTC	GCCCCCAGAGCCATACTGCT
**HNRNPF**	ACTGAAGGCATCCGTGGTTGT	GCCCAGCATCATGGACACTT
**HNRNPM**	TCCTGAGCGTCCACAACAACTT	TGGCATCAATGGGTTGCCCT
**IL1A**	GCAACCAACGGGAAGGTTCTGA	GCTCAGGAAGCTAAAAGGTGCTGA
**IL1B**	CTTGTTCTTTGAAGCTGATGGCCC	CCTTGCTGTAGTGGTGGTCGGA
**KHDRBS1**	CGCAGAACAAAGTTACGAAGGC	ATCTTGAACCTCCCCATGTCCA

**KHSRP**	AATCCGCAAGGACGCTTTCGC	CCCCAAAACCAAAATCAGGAGTGC
**L3MBTL2**	TGGTCTTGGGCTTCGACTGGG	AGCACCTCCACCTTCATCCCTT
**MDM4**	AACTACTGGGACGTCAGAGCTT	CTGTAGTAGCAGTGGCTAAAGTGAC
**NCF2**	GGAGGCACTCTTCAGTTATGAGGC	TTCCCTTTGCACTCCCCTTCCA
**PPIH**	ATTGGCTGGATGGGAAGCATGT	CTCAATCTTTCTCATCACTAGAAGTCCA
**PRPF4**	TGGGGGAACCCATCACACTTTT	AAGGCATCAGTACCGACAACTG
**PRPF8**	GCCCTCTAGCCCCGCTACC	TCCACAAACCCAAACTTCCGCT
**PTBP1**	CCGGGCTCCAAGAACTTCCAGA	ACGCCCCCATTGCTGGAAAAC
**RBM10**	TTGCAGGACGCTACACGATGGA	TGATCTTGGGCTTGGGGTCACT
**SERPING1**	CCAGGATATGCTCTCAATCATGGAGA	GCCTCCACCCCAGTCTCTGTC
**SF3A1**	GGAGGATTCTGCACCTTCTAA	GCGGTAGTAGGCATGGTAA
**SF3A2**	TGGCCCTGGAGACCATCGAC	TCATTGTTGTGAAGTGTCAGGCAGA
**SF3B1**	GTGGACAAAATGGCGAAGATCGC	GGCCCACTCCTTGAGCTTCATC
**SFRS7**	ATCGTTACAGCCGGCGAAGAA	TGCTGCGTGAGCGAGAGTAT
**SNRNP40**	TTGCCCCCAAAGAGAGATGTGT	TGCTTCCATCAGGTGACCAAGA
**SNRNP70**	CGAACCGTGAAGGGCTGGA	GCCTGAATGCCGGATGTTCAC
**SNRPA**	TCCATGCAGGGTTTCCCTTTCT	GAAGGTGCCTTTCATCTTGGCA
**SNRPA1**	GCAGGATGGTCAAGCTGACGG	GCATCAAACTGGTCTAACGTAGCACC
**SNRPB**	TAGGCCTCTGAGGAGGCGAAT	AGCATCTTGCTGCTCTTGCC
**SNRPB2**	TCCACAAATGCCTTGAGACAGCT	TCTTTGTCAGCAAAAGTTCCACGC
**SNRPF**	TGCTGTAGTCACGAGGGACGG	AGGAAAGGTTTGGGATTGAGGGGT
**SRSF1**	ATCCGAACCAAGGACATTGAGGAC	GCGTCTTCCGCGTCTCGC
**SRSF2**	CCGGAGCCGCAGCCCTA	TCGAGCGGCTGTAGCGAGAT
**SRSF3**	AGCTGATGCAGTCCGAGAGCT	ACCCCAAGAGGGAGGTGGGC
**TMEM199**	ACGCTTGGTTTCTTTCCGTCTCA	AGGCTTCACAACCTCTGGGAGA
**TRA2A**	AGCCGAAGCCATTCTCCAATGT	AGGCCAAACACTCCAAGGCA
**TRA2B**	AAGACCACATACGCCAACACCA	TCTGTCATAGTAATCCCGACGGC
**U2AF1**	AACGTCTGTGACAACCTGGGA	AGCCTTTTCCGCATCTTCCTCA
**VAMP7**	CCAGCCTCCTCTGGGAGCGG	GGCAACAACAGCAAAAAGAATCGC
**GADD45A**	GGCTGGAGAGCAGAAGACCGAA	GCTTCGTACACCCCGACAGTGA
**GDF15**	AGCTGGGAAGATTCGAACACCG	GGCCCGAGAGATACGCAGGT
